# The global epidemiology of bladder cancer: a joinpoint regression analysis of its incidence and mortality trends and projection

**DOI:** 10.1038/s41598-018-19199-z

**Published:** 2018-01-18

**Authors:** Martin C. S. Wong, Franklin D. H. Fung, Colette Leung, Wilson W. L. Cheung, William B. Goggins, C. F. Ng

**Affiliations:** 1 Division of Family Medicine and Primary Healthcare, School of Public Health and Primary Care, Faculty of Medicine, The Chinese University of Hong Kong, Prince of Wales Hospital, Shatin, New Territories, Hong Kong, China; 2 Family Medicine and Primary Health Care, Hospital Authority, Hong Kong, China; 3 Division of Biostatistics, School of Public Health and Primary Care, Faculty of Medicine, The Chinese University of Hong Kong, Hong Kong, China; 4 Division of Urology, Department of Surgery, Faculty of Medicine, The Chinese University of Hong Kong, Hong Kong, China

## Abstract

We tested the hypotheses that the global incidence of bladder cancer was increasing but its mortality was reducing and its incidence was positively correlated with country-specific socioeconomic development. We retrieved data on age-standardized incidence and mortality rates/100,000 from the GLOBOCAN database in 2012. Temporal patterns were examined for 39 countries from the Cancer Incidence in Five Continents volumes I-X and other national registries. We evaluated the correlation between the incidence/mortality rates and Human Development Index (HDI)/ logarithmic values of Gross Domestic Product per capita (GDP). The average annual percent change of the incidence and mortality rates in the most recent 10 years was examined by joinpoint regression analysis. The highest incidence rates were observed in Southern Europe, Western Europe and North America. The mortality rates were the highest in Western Asia and Northern Africa. The incidence was positively correlated with HDI (r = 0.66 [men]; r = 0.50 [women]) and to a lesser extent logarithmic values of GDP per capita (r = 0.60 [men]; r = 0.50 [women], all p < 0.01). Many European countries experienced incidence rise. A substantial mortality reduction was observed in most countries, yet increases in mortality rates were observed in the Philippines and Iceland. These findings identified countries where more preventive actions are required.

## Introduction

Bladder cancer was the ninth most common malignancy worldwide, with 430,000 newly diagnosed cases in 2012^1^. In Europe, a total of 118,000 new cases and 52,000 deaths were estimated in the same year^2^. Its high prevalence, in conjunction with its vulnerability to multiple recurrences and progression despite local therapy^3^, leads to a substantial health service burden^4^. Leal and colleagues^4^ recently estimated that bladder cancer cost €4.9 billion in the European Union in 2012. The majority (90%) of bladder cancer consists of urothelial carcinoma as the predominant histologic type in Western Europe and the United States, although squamous cell bladder cancer is more common in Africa where schistosomiasis infections were more prevalent^5^. Recent studies showed that North America and Western Europe reported particularly high incidence rates^6^, whilst Eastern Europe and Asian countries had the lowest rates.

The major risk factors for bladder cancer include tobacco smoking; industrial exposure to potential carcinogens such as aromatic amines and carbon black dust; long-term drinking of arsenic-contaminated or chlorinated water; and family history of concordant cancers^7,8^. Many of these risk factors can be modified by lifestyle measures and environmental protective initiatives, implying a strong prospect for intervention. Previous studies that analyzed its global trends were based on figures in the 1990s to early 2000s, did not make direct comparisons between countries, or performed in selected regions^8–11^. The Global Burden of Disease Study^12^ does provide comparison of bladder cancer incidence and mortality over time, but the temporal incidence and mortality trends of this cancer at the global level should be examined by recognized databases. Examining the patterns and temporal trends of bladder cancer could quantify geographical variations, shed light on health planning and priority setting, and explore modifiable factors that might have brought about trend changes^8–11^.

At least two important knowledge gaps exist in research on bladder cancer. Firstly, previous analyses showed that the highest incidence was found in more developed countries^1^, and the past few decades had seen wealth and technological advancement, particularly in more developed countries. One study showed that the age-specific incidence of papillary non-invasive bladder cancer increased from 5.52 to 9.09 per 100,000 from 1998 to 2006, but this is constrained to the US population^9^. Temporal changes in its trends of incidence and mortality for a significant number of countries in the past decade remain unknown. Also, there is a scarcity of studies on the role of socioeconomic development on incidence and mortality rate of bladder cancer when their associations were examined on a global scale. There was some evidence that environmental and socioeconomic factors affect bladder cancer mortality, and the effects appear to vary by gender and race^13^. A recent study has also examined the relationship between bladder cancer incidence/mortality and the world development index, but the influence of gender was not taken into account^14^. These findings highlight the need for a worldwide, across-country analysis of the epidemiological data.

This study tested the *a priori* hypothesis that the global temporal trends in the incidence of bladder cancer increased and that in its mortality decreased with time. Also, we sought to test the hypothesis that its global incidence was positively correlated with country-specific socioeconomic development.

## Methods

### Source of Data

To standardize the methodology across published literature, we adopted the same analysis plan as reported in our previous study on prostate cancer^15^, liver cancer^16^, pancreatic cancer^17^ and that on colorectal cancer^18^. We retrieved the incidence and mortality figures for bladder cancer in 2012 from the GLOBOCAN database^1^. For all countries, data were matched with their Human Development Index (HDI) and Gross Domestic Product (GPD) per capita in the same year based on the United Nations Human Development Report^19^, which highlighted the progress on human development over the past quarter century by reporting different statistical indexes. HDI is a summary index of life expectancy, education period, and income per capita^19^. For incidence trends, we extracted data from the *Cancer Incidence in Five Continents* series Volumes I-X^20^, which provided high-quality incidence statistics of cancer documented by local registries worldwide. This study has been approved by the Survey and Behavioural Research Ethics Committee of the Chinese University of Hong Kong. As this study used routinely collected anonymised electronic data consent was not required. All methods were performed in accordance with the relevant guidelines and regulations, and there were no publication of identifying information.

To acquire incidence data for more recent years, we also utilized publicly available information from the European Union Registration (EUREG)^21^, National Cancer Institute of the United States^22^, Nordic Cancer Registries^23^, Australian Cancer Incidence and Mortality Books^24^ and the Ministry of Health of New Zealand^25^. We used the GLOBOCAN data to analyze the incidence and mortality patterns in 2012, which were plotted against the HDI and logarithmic values of GDP per capita of each country in the same year. For analysis of temporal trends of incidence/mortality across time, we used the data from CI5 supplemented by the national databases^21–25^ (Table 1). The incidence data were retrieved according to the International Classification of Diseases (ICD-10, C67, 67.9; ICD-9-CM 188).

**Table 1:** Data source for the age-standardized incidence and mortality rates of bladder cancer.

	Incidence	Mortality
Austria	EUREG (1990–2009)	WHO (1980–2014)
Croatia	CI5 (1988–2007)	WHO (1985–2013)
Czech Republic	CI5 (1983–2007)	WHO (1986–2013)
Denmark	NORDCAN (1960–2013)	NORDCAN (1960–2013)
Estonia	CI5 (1968–2007)	WHO (1994–2012)
Finland	NORDCAN (1960–2013)	NORDCAN (1960–2013)
France	CI5 (1988–2007)	WHO (1979–2011)
Germany	CI5 (1970–2007)	WHO (1990–2013)
Iceland	NORDCAN (1960–2013)	NORDCAN (1960–2012)
Italy	CI5 (1993–2007)	WHO (1979–2003, 2006–2012)
Latvia	CI5 (1988–2007)	WHO (1996–2012)
Lithuania	CI5 (1978–2007)	WHO (1993–2013)
Netherlands	CI5 (1989–2007)	WHO (1979–2013)
Norway	NORDCAN (1960–2013)	NORDCAN (1960–2013)
Poland	CI5 (1978–2006)	WHO (1980–1996, 1999–2013)
Slovakia	CI5 (1968–2007)	WHO (1992–2010, 2012–2014)
Slovenia	CI5 (1963–2007)	WHO (1985–2010)
Spain	CI5 (1993–2007)	WHO (1980–2013)
Sweden	NORDCAN (1960–2013)	NORDCAN (1960–2013)
Switzerland	CI5 (1993–2007)	WHO (1995–2013)
United Kingdom	CI5 (1993–2007)	WHO (1979–2013)
Australia	AIHW (1982–2012)	AIHW (1968–2013)
New Zealand	New Zealand (1960–2012)	New Zealand (1960–2012)
Bulgaria	EUREG (1993–2007)	WHO (1980–2012)
Russia	CI5 (1994–2007)	WHO (1999–2011)
Malta	EUREG (1994–2009)	EUREG (1995–2010)
Ireland	EUREG (1994–2009)	WHO (1979–2012)
Brazil	CI5 (1988–2007)	WHO (1979–2013)
Colombia	CI5 (1983–2007)	WHO (1984–2012)
Costa Rica	CI5 (1980–2007)	WHO(1980–2013)
Ecuador	CI5 (1985–2007)	WHO (1979–2013)
Canada	CI5 (1978–2007)	WHO (1979–2011)
USA	NIH (1975–2013)	NIH (1975–2013)
USA White	NIH (1975–2013)	NIH (1975–2013)
USA Black	NIH (1975–2013)	NIH (1975–2013)
Israel	CI5 (1963–2007)	WHO (1979–2013)
Japan	CI5 (1988–2007)	WHO (1979–2013)
Philipines	CI5 (1983–2007)	WHO (1992–2003, 2008)
Singapore	CI5 (1968–2007)	WHO (1979–2014)
Thailand	CI5 (1993–2007)	WHO (1979–1987, 1990–1992, 1994–2000, 2002–2006)
China	CI5 (1993–2007)	Hospital Authority (1983–2013)

AIHW: Australian Cancer Incidence and Mortality Books^24^; CI5: Cancer Incidence in Five Continents V^20^; EUREG: European Union Registration^21^; Hospital Authority, Hong Kong. http://www3.ha.org.hk/cancereg/e_a1b.asp; New Zealand: the Ministry of Health of New Zealand^25^; NIH: National Institute of Health, United States^22^; NORDCAN: Nordic Cancer Registries^23^; WHO: World Health Organization^26^.

For mortality data, we used the WHO mortality database^26^ and the various national databases^21–23^, where the primary data source originated from death certificates. These data were categorized based on the ICD 9^th^ 188 up to 2014. Table 1 showed a more detailed description of the data sources and calendar years for the present analysis. We used age-standardized rate per 100,000 (ASR) using the world standard population^27^. More developed regions include Europe, Northern America, Australia/New Zealand and Japan, whilst less developed regions include Africa, Asia (excluding Japan), Latin America and the Caribbean, Melanesia, Micronesia and Polynesia^1^.

### Statistical Analysis

We used joinpoint regression analysis to study the incidence and mortality trends^28^. A series of joined straight lines was fit to the ASR trend^28^. We performed logarithmic transformation of the ASRs and computed the standard errors adopting binomial approximation. A maximum number of three joinpoints were used as analysis options, and we evaluated the average annual percent change (AAPC) and the respective 95% confidence intervals (C.I.) for data available in the most recent 10 years. The AAPC was computerized as a geometrically weighted average of the generated APCs by the joinpoint trend analysis software. Their weights were equivalent to the length of each segment within the specified time interval^29^. We extracted data for the incidence and mortality trends from the above sources. We selected the most recent 10 years as the timeframe for examining temporal trend changes, as this was commonly adopted in previous studies on global epidemiology of other cancers^15,18,30^. All AAPCs with their 95% C.I. lying above and below zero, respectively, were regarded as increasing and decreasing trends. AAPCs with 95% C.Is overlapping with zero were considered as stable trends^15,18,30^. We plotted the ASRs of incidence and mortality against the HDI and logarithmic values of GDP per capita, respectively, for each country. The HDI was defined as low ( ≤ 0.534), medium (0.534–0.710), high (0.710–0.796) and very high (>0.796)^19^. Linear regression analysis was applied and correlation coefficients were evaluated, as linear associations had the best goodness-of-fit. Also, we estimated the percent change in incidence and mortality by 2020 and 2030 when compared to the latest published figures based on the AAPC – a statistical technique employed by Bailey and colleagues in a recent article published in *JAMA Surgery*^31^. The predicted incidence/mortality rates were assumed to be a constant percentage of the rate of the previous joinpoint. All p values <0.05 were considered statistically significant.

## Results

### Incidence and mortality rates of bladder cancer in 2012

A total of 429,793 new cases of bladder cancer and 165,084 related deaths were reported in 2012 (Tables 2 and 3). The age-standardized rate of its incidence showed approximately ten-fold variation worldwide^1^. In men, the highest rates were found in Southern Europe (ASR = 21.8), Western Europe (ASR = 19.7), North America (ASR = 19.5) and Western Asia (ASR = 19.0), and the lowest were reported in Western (ASR = 2.1), Middle (ASR = 2.2) and Eastern Africa (ASR = 3.3). This geographical difference is similar for incidence rates in women. Overall, countries that were more developed had higher incidence than less developed regions in both genders. The incidence in men was substantially higher in countries with very high HDI (ASR = 16.7) than those with high (ASR = 10.8), medium (ASR = 4.7) and low HDI (ASR = 3.1), and similarly this trend was also observed for women (Supplementary Figure 1).

**Table 2:** The estimated incidence and mortality of bladder cancer according to world area, 2012, males.

World regions	Population size Male (thousands)	New cases		Mortality		Mortality to incidence ratio
		n	ASR	n	ASR	
**Africa**	549,445	17685	6.3	9362	3.5	0.56
Eastern Africa	180,243	2824	3.3	1819	2.2	0.67
Middle Africa	69,179	610	2.2	420	1.6	0.73
Northern Africa	106,147	11225	15.1	5489	7.6	0.50
Southern Africa	29,735	1285	7.5	494	3.0	0.40
Western Africa	164,141	1741	2.1	1140	1.5	0.71
**Asia**	2,179,003	115646	5.5	52816	2.5	0.45
Eastern Asia	813,296	64662	5.8	27271	2.3	0.40
South-Eastern Asia	305,225	10784	4.3	5352	2.2	0.51
South-Central Asia	933,786	24415	3.6	13413	2.0	0.56
Western Asia	126,697	15785	19.0	6780	8.4	0.44
**America**	303,514	17610	6.1	7078	2.4	0.39
Caribbean	20,951	1839	7.6	773	3.0	0.39
Central America	82,227	2327	3.4	867	1.2	0.35
South America	200,336	13444	6.9	5438	2.7	0.39
North America	173,209	58089	19.5	13285	4.0	0.21
**Europe**	355,275	118365	17.7	39522	5.2	0.29
Central and Eastern Europe	138,249	30871	15.1	13231	6.1	0.40
Northern Europe	49,574	12722	12.4	5174	4.4	0.35
Southern Europe	74,900	34786	21.8	11653	6.0	0.28
Western Europe	92,553	39986	19.7	9464	4.0	0.20
**Oceania**	18,859	2985	10.6	988	3.2	0.30
Australia/New Zealand	13,632	2868	11.3	939	3.3	0.29
Melanesia	4,628	84	3.5	43	2.0	0.57
Micronesia/Polynesia	258	33	6.5	6	1.2	0.18
More developed regions	604,008	196077	16.9	58914	4.5	0.27
Less developed regions	2,975,297	134303	5.3	64137	2.6	0.49
**World**	3,579,305	330380	9.0	123051	3.2	0.36

ASR = Age standardized rate per 100,000. Source: GLOBOCAN 2012^1^. Numbers are rounded to the nearest 10 or 100, and may not add up to the total. The population size of the world regions were retrieved from the Population Reference Bureau, Washington, DC. Available at: http://www.prb.org/Publications/Datasheets/2012/world-population-data-sheet/world-map.aspx#/table/population.

**Table 3:** The estimated incidence and mortality of bladder cancer according to world area, 2012, Females.

World regions	Population size Female (thousands)	New cases		Mortality		Mortality to incidence ratio
		n	ASR	n	ASR	
**Africa**	549 608	6752	2.1	3906	1.2	0.57
Eastern Africa	182 469	1961	2.0	1290	1.3	0.65
Middle Africa	69 644	441	1.3	300	0.9	0.69
Northern Africa	105 353	2708	3.2	1337	1.6	0.50
Southern Africa	30 816	483	1.9	225	0.9	0.47
Western Africa	161 327	1159	1.3	754	0.9	0.69
**Asia**	2 081 150	32922	1.4	16478	0.6	0.43
Eastern Asia	777 374	20789	1.6	10220	0.7	0.44
South-Eastern Asia	306 008	3034	1.0	1517	0.5	0.50
South-Central Asia	881 514	6159	0.8	3441	0.5	0.63
Western Asia	116 253	2940	3.1	1300	1.3	0.42
**America**	310 360	7234	2.0	3069	0.8	0.40
Caribbean	21 313	542	1.8	284	0.9	0.50
Central America	83 632	1430	1.8	535	0.6	0.33
South America	205 415	5262	2.1	2250	0.9	0.43
North America	176 585	18660	5.1	5307	1.2	0.24
**Europe**	381 747	32932	3.5	12889	1.1	0.31
Central and Eastern Europe	155 701	8904	2.7	3543	0.9	0.33
Northern Europe	51 252	4645	3.6	2391	1.5	0.42
Southern Europe	78 393	8049	3.8	3001	1.0	0.26
Western Europe	96 400	11334	4.3	3954	1.1	0.26
**Oceania**	18 746	913	2.7	384	1.0	0.37
Australia/New Zealand	13 715	887	2.9	366	1.0	0.34
Melanesia	4 451	21	0.7	13	0.4	0.57
Micronesia/Polynesia	580	5	0.9	5	0.9	1.00
More developed regions	637 294	57766	3.7	21024	1.1	0.30
Less developed regions	2 880 901	41647	1.5	21009	0.7	0.47
**World**	3 518 195	99413	2.2	42033	0.9	0.41

ASR = Age standardized rate per 100,000. Source: GLOBOCAN 2012^1^. Numbers are rounded to the nearest 10 or 100, and may not add up to the total. The population size of the world regions were retrieved from the Population Reference Bureau, Washington, DC. Available at: http://www.prb.org/Publications/Datasheets/2012/world-population-data-sheet/world-map.aspx#/table/population.

The mortality rates varied by seven-fold in 2012, and were higher in more developed than less developed regions (ASR = 4.5 vs. 2.6 in men; 1.1 vs. 0.7 in women). The highest mortality rates in men were reported in Western Asia (ASR = 8.4), Northern Africa (ASR = 7.6), and Central and Eastern Europe (ASR = 6.1). The lowest estimated death rates were found in Central America (ASR = 1.2), Micronesia/Polynesia (ASR = 1.2), Western Africa (ASR = 1.5), and Middle Africa (ASR = 1.6). In women, Northern Europe was amongst one of the world regions with the highest mortality (ASR = 1.5), whilst South-Eastern and South-Central Asia (ASR = 0.5) were regions that reported the lowest mortality rates.

### Correlation between incidence/mortality and socioeconomic development

**Figure 1:**
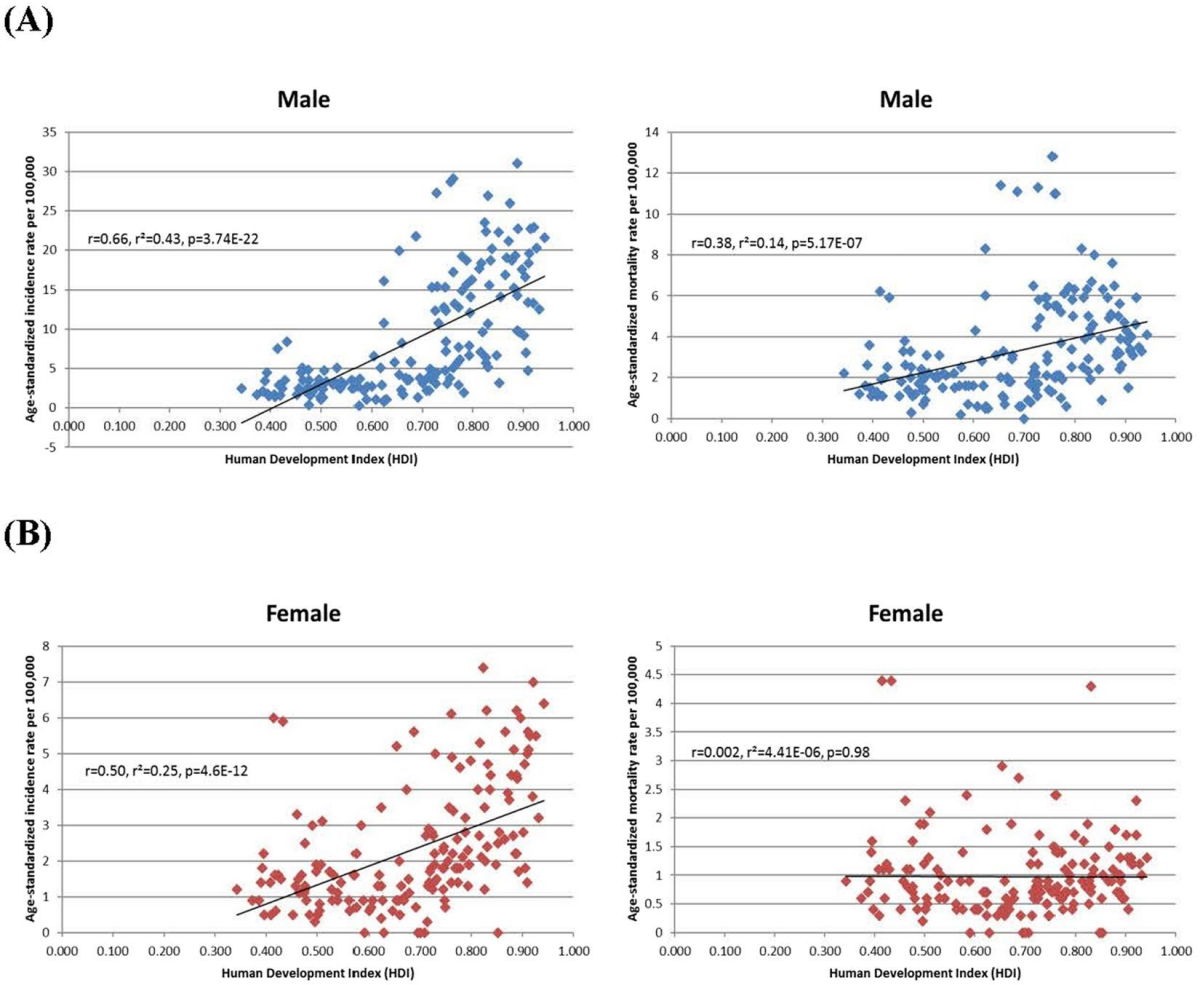
(**A**) Correlation between age-standardised bladder cancer incidence (left panel) and mortality (right panel) and Human Development Index (HDI) (Male) (**B**) Correlation between age-standardised bladder cancer incidence (left panel) and mortality (right panel) and Human Development Index (HDI) (Female).

**Figure 2:**
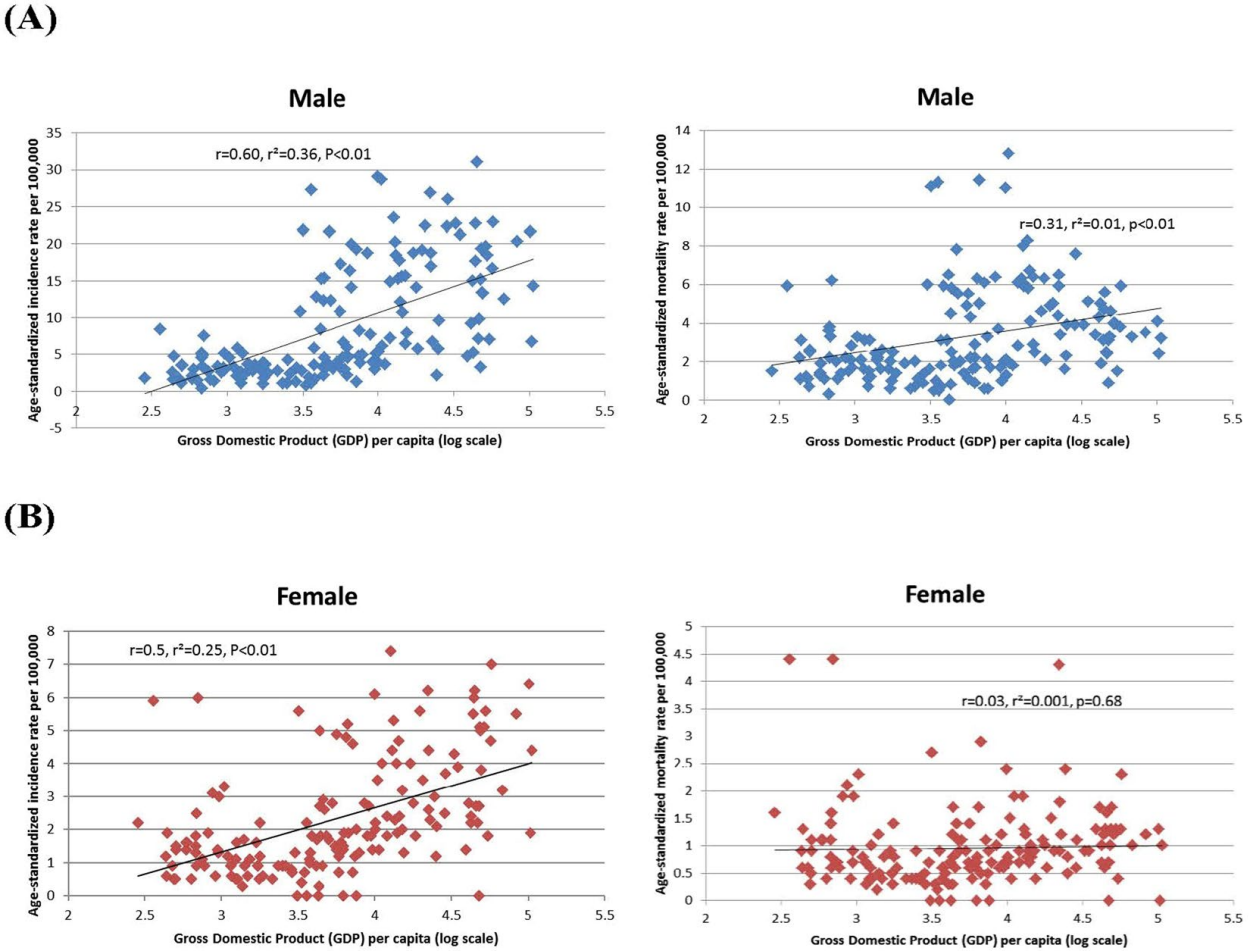
(**A**) Correlation between age-standardised bladder cancer incidence (left panel) and mortality (right panel) and logarithmic values of Gross Domestic Product (GDP) (Male) (**B**) Correlation between age-standardised bladder cancer incidence (left panel) and mortality (right panel) and logarithmic values of Gross Domestic Product (GDP) (Female).

Figures 1A,B and 2A,B showed the correlation between the incidence/mortality and the two socioeconomic indicators, evaluated by simple linear regression analysis. The ASR of incidence in men (r = 0.66, r^2^ = 0.43, p < 0.001) and women (r = 0.50, r^2^ = 0.25, p < 0.001) increased with higher levels of HDI, and to a lesser extent logarithmic values of GDP per capita (r = 0.60, r^2^ = 0.36, p < 0.01 [men] and r = 0.50, r^2^ = 0.25, p < 0.01 [women]). The ASR of mortality in women was not significantly correlated with HDI and logarithmic values of GDP per capita, whereas that in men was significantly correlated with HDI (r = 0.38, r^2^ = 0.14, p < 0.001) and logarithmic values of GDP per capita (r = 0.31, r^2^ = 0.01, p < 0.01).

### Temporal trends of bladder cancer

**Figure 3:**
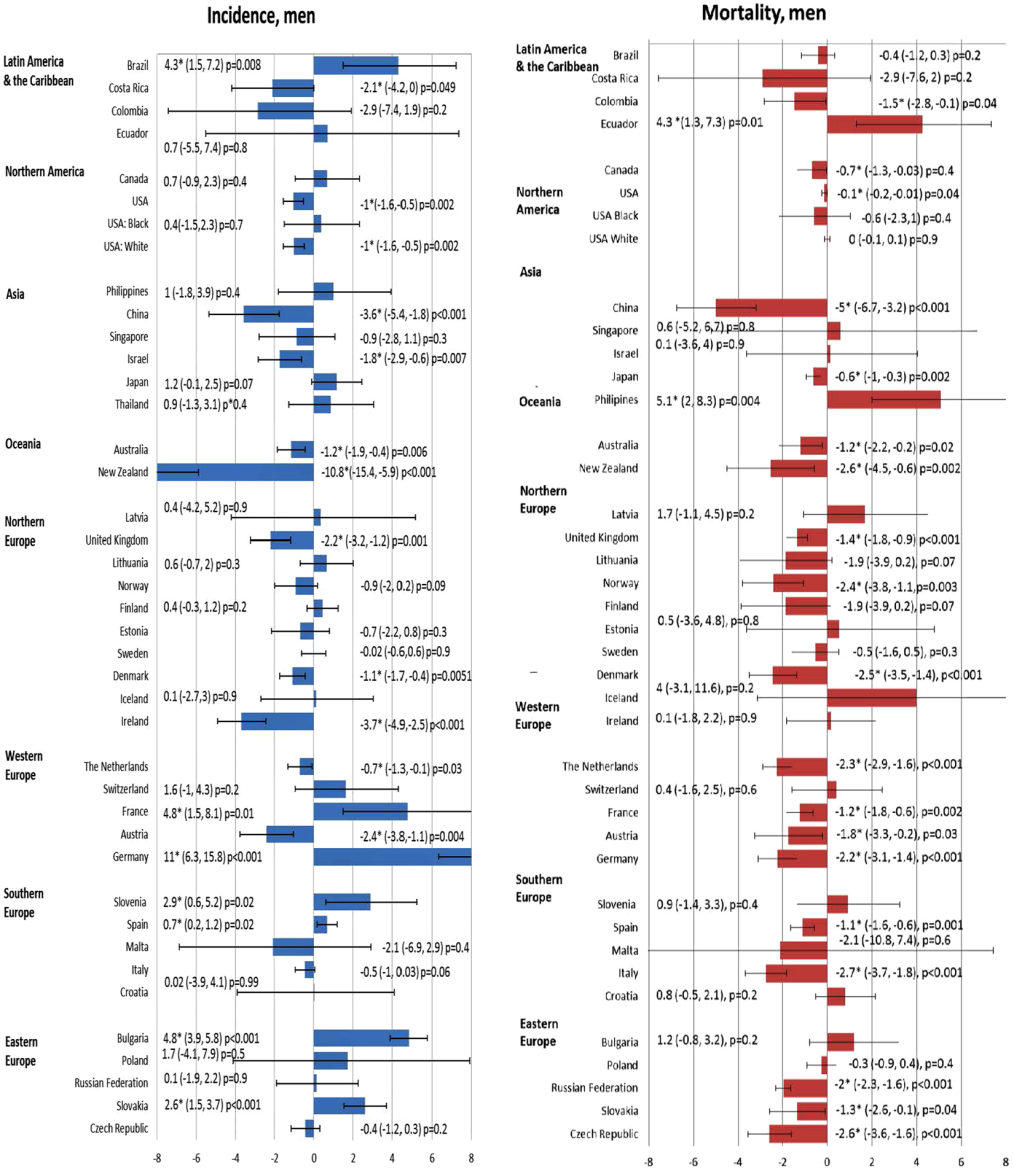
The Average Annual Percent Change (AAPC) in the incidence of bladder cancer in male (left) and female (right) in the most recent 10 years.

**Figure 4:**
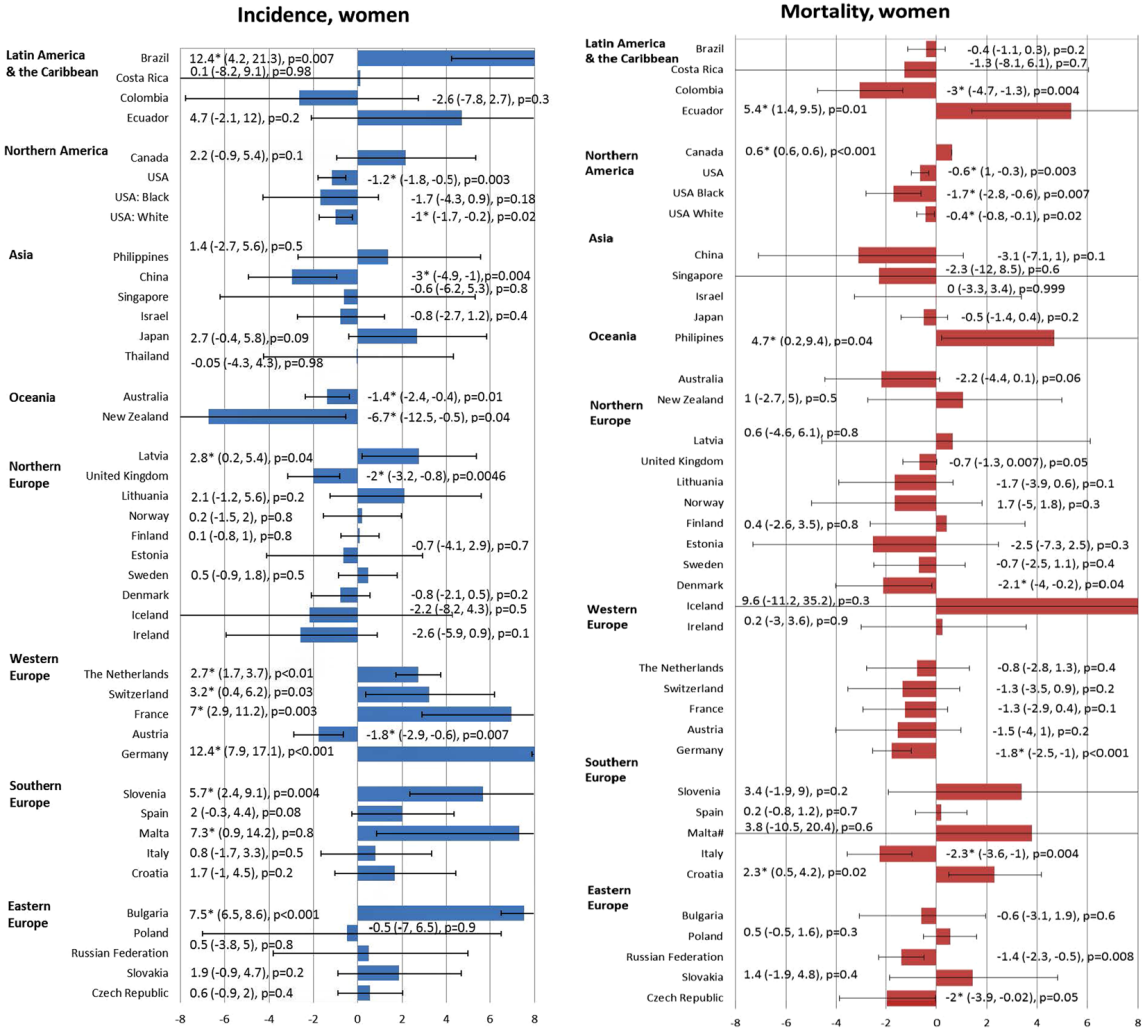
The Average Annual Percent Change (AAPC) in the incidence of bladder cancer in male (left) and female (right) in the most recent 10 years.

The incidence and mortality trends of each country were shown in Supplementary Figure 1, and the corresponding findings from the joinpoint regression analysis were presented in Supplementary Figures 2 and 3. The changes in incidence and mortality trends were plotted in Figs 3 and 4.

### Incidence trend

Among men, seven countries had increases in incidence, 11 countries showed declining trends, and 21 countries reported stable trends. Out of all seven countries with rise in incidence, six were reported in Europe, including Germany (AAPC = 11.0, 95% C.I. 6.3, 15.8, p < 0.001), Bulgaria (AAPC = 4.8, 95% C.I. 3.9, 5.8, p < 0.001), France (AAPC = 4.8, 95% C.I. 1.5, 8.1, p = 0.01), Slovenia (AAPC = 2.9, 95% C.I. 0.6, 5.2, p = 0.02) and Slovakia (AAPC = 2.6, 95% C.I. 1.5, 3.7, p < 0.001). Countries with substantial incidence reduction include New Zealand (AAPC = −10.8, 95% C.I. −15.4, −5.9, p < 0.001), Ireland (AAPC = −3.7, 95% C.I. −4.9, −2.5, p < 0.001) China (AAPC = −3.6, 95% C.I. −5.4, −1.8, p < 0.001) and Austria (AAPC = −2.4, 95% C.I. −3.8, −1.1, p = 0.004). Among women, seven countries had increases in incidence, 8 countries showed declining trends, and trends in 24 countries remained stable. The majority of incidence rise occurred in Europe, and New Zealand also reported a significant incidence decline (AAPC = −6.7, 95% C.I. −12.5, −0.5, p = 0.04) (Fig. 4).

### Mortality trend

In men, the only countries that showed increasing mortality trends include Ecuador (AAPC = 4.3, 95% C.I. 1.3, 7.3, p = 0.01) and the Philippines (AAPC = 5.1, 95% C.I. 2.0, 8.3, p = 0.004). A total of 19 out of 38 countries reported declining trends, and 12 occurred in European countries. Among them, Italy (AAPC = −2.7, 95% C.I. −3.7, −1.8, p < 0.001), Czech Republic (AAPC = −2.6, 95% C.I. −3.6, −1.6, p < 0.001), Denmark (AAPC = −2.5, 95% C.I. −3.5, −1.4, p < 0.001) and Norway (AAPC = −2.4, 95% C.I. −3.8, −1.1, p = 0.003) showed the most marked reduction in mortality rates.

In women, Ecuador (AAPC = 5.4, 95% C.I. 1.4, 9.5, p = 0.01), the Philippines (AAPC = 4.7, 95% C.I. 0.2, 9.4, p = 0.04), Croatia (AAPC = 2.3, 95% C.I. 0.5, 4.2, p = 0.02) and Canada (AAPC = 0.6, 95% C.I. 0.6, 0.6, p < 0.001) were the only countries where increase in mortality rates were observed. Colombia had the greatest mortality decline (AAPC = −3.0, 95% C.I. −4.7, −1.3, p = 0.004). Five European countries reported decrease in mortality trends, including Italy (AAPC = −2.3, 95% C.I. −3.6, −1.0, p = 0.004), Denmark (AAPC = −2.1, 95% C.I. −4.0, −0.2, p = 0.04), Czech Republic (AAPC = −2.0, 95% C.I. −3.9, −0.02, p = 0.05), Germany (AAPC = −1.8, 95% C.I. −2.5, −1.0, p < 0.001) and Russian Federation (AAPC = −1.4, 95% C.I. −2.3, −0.5, p = 0.008).

### Projection of incidence and mortality rates in 2020 and 2030

Tables 4 and 5 summarized the projected incidence and mortality of bladder cancer by all future years up to 2030. The estimated increase in incidence by 2030 was most marked in Germany (998%), France (191%), Bulgaria (129%), and Brazil (164%) among men. In women, Brazil (1,380%), Germany (1,375%), Bulgaria (372%) and France (371%) were projected to have the highest incidence growth. Most countries included in this study showed declining mortality rates, except the Philippines (283%) and Ecuador (104%) that showed increase rates in men. Among women, Iceland (374%), the Philippines (245%), Ecuador (143%) and Malta (111%) showed prominent rise in mortality rates.

**Table 4:** Projected growth rate of bladder cancer incidence rates by 2020 and 2030.

Men						Women				
	AAPC	P-value (3 sig. fig.)	Latest available year*	Growth rate by 2020 (%)	Growth rate by 2030 (%)	AAPC	P-value	Latest available year*	Growth rate by 2020 (%)	Growth rate by 2030 (%)
**Latin America, the Caribbean and Northern America**										
Brazil	4.31	0.008	2007	73	164	12.43	0.007	2007	359	1,380
Costa Rica	−2.13	0.050	2007	−24	−39	0.11	0.979	2007	1	3
Colombia	−2.87	0.200	2007	−32	−49	−2.65	0.284	2007	−29	−46
Ecuador	0.71	0.804	2007	10	18	4.73	0.152	2007	82	189
Canada	0.68	0.366	2007	9	17	2.16	0.149	2007	32	63
**Asia**										
The Philippines	1.01	0.437	2007	14	26	1.36	0.465	2007	19	37
China	−3.59	0.000	2007	−38	−57	−2.96	0.004	2007	−32	−50
Singapore	−0.89	0.323	2007	−11	−19	−0.61	0.837	2007	−8	−13
Israel	−1.75	0.007	2007	−21	−33	−0.78	0.386	2007	−10	−17
Japan	1.16	0.069	2007	16	30	2.67	0.090	2007	41	83
Thailand	0.87	0.381	2007	12	22	−0.049	0.979	2007	−1	−1
**Oceania**										
Australia	−1.11	0.008	2012	−9	−18	−1.23	0.032	2012	−9	−20
New Zealand	−10.80	0.000	2012	−60	−87	−6.74	0.037	2012	−43	−71
**Northern Europe**										
Latvia	0.36	0.879	2007	5	9	2.76	0.038	2007	42	87
United Kingdom	−2.22	0.001	2007	−25	−40	−1.99	0.005	2007	−23	−37
Lithuania	0.65	0.344	2007	9	16	2.11	0.187	2007	31	62
Norway	−0.92	0.091	2013	−6	−15	0.20	0.801	2013	1	3
Finland	0.44	0.237	2013	3	8	0.094	0.805	2013	1	2
Estonia	−0.70	0.306	2007	−9	−15	−0.66	0.678	2007	−8	−14
Sweden	−0.019	0.944	2013	0	0	0.46	0.445	2013	3	8
Denmark	−1.09	0.005	2013	−7	−17	−0.78	0.248	2013	−5	−13
Iceland	0.12	0.926	2013	1	2	−2.17	0.451	2013	−14	−31
Ireland	−3.70	0.000	2009	−34	−55	−2.58	0.123	2009	−25	−42
**Western Europe**										
The Netherlands	−0.72	0.028	2007	−9	−15	2.74	0.000	2007	42	86
Switzerland	1.64	0.220	2007	24	45	3.24	0.031	2007	51	108
France	4.76	0.010	2007	83	191	6.97	0.004	2007	140	371
Austria	−2.42	0.004	2009	−24	−40	−1.77	0.007	2009	−18	−31
Germany	10.98	0.001	2007	287	998	12.41	0.000	2007	358	1375
**Southern Europe**										
Slovenia	2.89	0.019	2007	45	92	5.67	0.004	2007	105	256
Spain	0.68	0.015	2007	9	17	2.02	0.076	2007	30	59
Malta	−2.10	0.356	2009	−21	−36	7.32	0.031	2009	118	341
Italy	−0.46	0.065	2007	−6	−10	0.80	0.479	2007	11	20
Croatia	0.016	0.993	2007	0	0	1.68	0.194	2007	24	47
**Eastern Europe**										
Bulgaria	3.67	0.000	2007	60	129	6.98	0.000	2007	140	372
Poland	1.72	0.524	2006	27	51	−0.48	0.875	2006	−6	−11
Russian Federation	0.15	0.872	2007	2	4	0.50	0.801	2007	7	12
Slovakia	2.61	0.000	2007	40	81	1.87	0.157	2007	27	53
Czech Republic	−0.43	0.213	2007	−5	−9	0.56	0.398	2007	8	14

AAPC: Average Annual Percent Change; *represents the calendar year where the latest figure is available from the respective national database.

**Table 5:** Projected growth rate of bladder cancer mortality rates by 2020 and 2030.

Men						Women				
	AAPC	P-value (3 sig. fig.)	Latest available year*	Growth rate by 2020 (%)	Growth rate by 2030 (%)	AAPC	P-value	Latest available year*	Growth rate by 2020 (%)	Growth rate by 2030 (%)
**Latin America, the Caribbean and Northern America**										
Brazil	−0.42	0.228	2013	−3	−7	−0.40	0.244	2013	−3	−7
Costa Rica	−2.92	0.201	2013	−19	−40	−1.27	0.692	2013	−9	−19
Colombia	−1.46	0.043	2012	−11	−23	−3.05	0.004	2012	−22	−43
Ecuador	4.28	0.010	2013	34	104	5.37	0.014	2013	44	143
Canada	−0.69	0.042	2011	−6	−12	0.60	0.000	2011	6	12
**Asia**										
China	−4.98	0.000	2013	−30	−58	−3.10	0.121	2013	−20	−41
Singapore	0.58	0.826	2014	4	10	−2.29	0.622	2014	−13	−31
Israel	0.14	0.937	2013	1	2	−0.0017	0.999	2013	0	0
Japan	−0.63	0.002	2013	−4	−10	−0.50	0.244	2013	−3	−8
The Philippines	5.10	0.005	2003	133	283	4.69	0.043	2003	118	245
**Oceania**										
Australia	−1.21	0.025	2013	−8	−19	−2.11	0.069	2013	−14	−30
New Zealand	−2.56	0.017	2012	−19	−37	1.05	0.547	2012	9	21
**Northern Europe**										
Latvia	1.67	0.201	2012	14	35	0.63	0.792	2012	5	12
United Kingdom	−1.36	0.000	2013	−9	−21	−0.67	0.052	2013	−5	−11
Lithuania	−1.89	0.071	2013	−12	−28	−1.66	0.133	2013	−11	−25
Norway	−2.44	0.003	2013	−16	−34	−1.65	0.298	2013	−11	−25
Finland	−1.87	0.068	2013	−12	−27	0.39	0.777	2013	3	7
Estonia	0.50	0.791	2012	4	9	−2.53	0.270	2012	−19	−37
Sweden	−0.55	0.263	2013	−4	−9	−0.70	0.399	2013	−5	−11
Denmark	−2.46	0.001	2013	−16	−34	−2.11	0.036	2013	−14	−30
Iceland	3.98	0.240	2013	31	94	9.59	0.344	2013	90	374
Ireland	−0.06	0.960	2012	−1	−1	0.22	0.879	2012	2	4
**Western Europe**										
The Netherlands	−2.26	0.000	2013	−15	−32	−0.77	0.410	2013	−5	−12
Switzerland	0.42	0.639	2013	3	7	−1.34	0.203	2013	−9	−20
France	−1.24	0.002	2011	−11	−21	−1.26	0.145	2011	−11	−21
Austria	−1.76	0.029	2014	−10	−25	−1.55	0.191	2014	−9	−22
Germany	−2.23	0.000	2013	−15	−32	−1.77	0.001	2013	−12	−26
**Southern Europe**										
Slovenia	0.92	0.379	2010	10	20	3.39	0.182	2010	40	95
Spain	−1.12	0.001	2013	−8	−17	0.18	0.695	2013	1	3
Malta	−2.13	0.609	2010	−19	−35	3.80	0.571	2010	45	111
Italy	−2.74	0.000	2003	−38	−53	−2.27	0.004	2003	−32	−46
Croatia	0.80	0.202	2013	6	15	2.30	0.020	2013	17	47
**Eastern Europe**										
Bulgaria	2.09	0.061	2012	18	45	−0.60	0.599	2012	−5	−10
Poland	−0.26	0.393	2013	−2	−4	0.53	0.274	2013	4	9
Russian Federation	−1.98	0.000	2011	−16	−32	−1.39	0.008	2011	−12	−23
Slovakia	−1.34	0.038	2010	−13	−24	1.42	0.353	2010	15	32
Czech Republic	−2.60	0.000	2013	−17	−36	−1.97	0.048	2013	−13	−29

AAPC: Average Annual Percent Change; *represents the calendar year where the latest figure is available from the respective national database.

## Discussion

This study presented the most updated global epidemiological profiles of bladder cancer, and we described the incidence and mortality patterns and trends based on high quality data. Southern Europe, Western Europe, Northern America and Western Asia reported the highest incidence rates, whilst Western, Middle and Eastern Africa had the lowest rates. In men, the highest mortality rates were observed in Western Asia, Northern Africa, and Central and Eastern Europe. with lowest death rates found in Central America, Micronesia/Polynesia, Western Africa, and Middle Africa. In women, high mortality rates were observed in Northern Europe, whilst South-Eastern and South-Central Asia reported the lowest mortality rates. Incidence rates were positively correlated with human development levels and logarithmic values of GDP per capita with high coefficients of correlation between incidence/mortality and HDI was moderately strong (0.50–0.66). Incidence figures from 39 countries in the most recent 10 years reported that a total of 7 countries experienced increases in incidence rates in either gender - and six of them were European countries. Up to 19 and 6 out of 38 countries reported reduction in mortality trends in men and women, respectively, and the majority of them were in Europe. Bulgaria is the only country where males had significant increase in both incidence and mortality. On the contrary, male populations in Oceania (i.e. Australia and New Zealand) went in declines in both incidence and mortality. German men and women reported increases in incidence but decreases in mortality. A reduction in both incidence and mortality in both genders could only be observed in USA.

A recent study by Antoni *et al*.^32^ has examined the most recent global incidence and mortality patterns and trends of bladder cancer. They also reported the highest incidence rates in Southern and Western Europe, North America as well as in some nations in Northern Africa and Western Asia. It was found that the incidence rates were diverging, with stabilizing or decreasing rates for men but increasing rates for women in many countries. Our study utilized an approach similar to their design by using similar data sources, but the timeframe used to compute the AAPC and the registries used to extract the data were different.

Several reasons could explain the higher incidence of bladder cancer in more developed countries, and their positive correlation with HDI. Firstly, in developed nations with more rapid development and higher productivity, the prevalence of risk factors for bladder cancer including smoking, obesity, alcohol drinking and red-meat consumption was higher^7,8^ – and these risk factors have been reported by the World Health Organization as alarmingly high across Europe^33^. Tobacco use in developing nations is growing and has now surpassed that of developed nations where prevalence of tobacco use has begun to decrease^34^. To control it, the WHO FCTC (Framework Convention on Tobacco Control) were adopted by 174 countries and currently covering around 90% of the population globally^35^. There has been substantial interest in measuring the impact of tobacco use on health outcomes at the population level since the 1950s^36^. However, there are substantial differences between various surveys and a lag in the impact of tobacco control on smoking-related incidence and mortality^37^. While limited data was available to evaluate how the tobacco control has influenced the incidence or mortality of bladder cancer. In 2012, the WHO published the “WHO global report: mortality attributable to tobacco”, showing the relative risk (males: 3.0, females: 2.4) for bladder cancer death due to tobacco use was just second to lung cancer^38^. Therefore, we can predict that the policies of tobacco control will have a substantial impact on the incidence and mortality of bladder cancer. Another explanation for the higher incidence could be attributed to the more widespread practice of diagnostic tests in more resource-privileged countries, such as urine cytology, cystoscopy and CT scan for haematuria and other non-specific urinary symptoms presented by patients^39^. Yet another possible contributor includes higher rates of occupational and environmental exposures to carcinogenic agents, including the aromatic amines in the dye industry in the European Union^40^. Our findings that some countries outside Europe and North America reported markedly increased incidence trends warrant further studies to elucidate the underlying etiological mechanisms. The reduction in mortality trends in the recent decade could be explained by decreasing incidence or earlier diagnosis leading to stage migration to earlier stage disease, which could be treated by curative intervention. Another driver for the mortality decline could be due to improved endoscopic system for cystoscopic surveillance^41^, use of re-staging (second look) transurethral bladder resection^42^ and also better intravesical therapy for non-muscle invasive cancer^43^.

This study presented and analyzed the most up-to-date epidemiological data on bladder cancer, and quantified the geographical variations as well as trends in its incidence and mortality using data of high validity, completeness and comparability. We also used national mortality data that were based on WHO criteria of at least medium level quality in terms of coverage and completeness. The IARCs estimation methods have been further refined in more recent years to take into account the increasing availability and quality of the source data^1^. However, some limitations of this study should be discussed. Firstly, cancer registration in relatively less-developed nations could suffer from higher chance of under-reporting. Population based cancer registries that only cover parts of a country can have limited representativeness for the whole population. This is especially true for many population based cancer registries in low- and middle income countries that are mostly located in urban areas. On the contrary, in countries where estimates were based on a single cancer registry in more urbanized, resource privileged areas, the presented figures could be an overestimation if the countries consist of extensive rural populations. In addition, analysis of bladder cancer incidence is affected by the underlying data – some might include only invasive bladder cancer and others might include both invasive and non-invasive bladder cancer. Different cancer registries have different policies on how the cancer is reported, and this could change over time. Also, information regarding the tissue type of bladder cancer, such as urothelial cancer and squamous cell carcinoma, was lacking. Morover, despite our most inclusive approach to analyze the most recent data, the figures used are from 2012 at the latest and the temporal trends will need continuous updates. Last but not least, the change in coding practice of bladder cancer might influence the comparison of incidence/mortality trend of this disease across years. For instance, since 2000, the definition of bladder carcinoma was coded as invasive disease (ICD-10, C67), and it led to an apparent decrease in the incidence of bladder carcinoma as well as the survival rate due to the exclusion of carcinoma in situ that bears better prognosis. Although after years of efforts, the definition has become more universal across the cancer registries of different countries in recent years, there was a period around year 2000 with a mixed definition, which made the comparison of incidence/mortality rates among different registry systems difficult. Future analysis should be performed before and after this time period to capture the changes in trends more accurately.

## Conclusion

The incidence rates of bladder cancer increased in many European countries analyzed in this study, and the mortality rates declined in a large number of nations, particularly in more developed regions. With population ageing and population growth, the absolute incidence of bladder cancer might be further escalating in European nations. Appropriate healthcare resources should be allocated to cope with the increasing need for patient treatment. Future studies are needed to explore the underlying mechanisms for these epidemiological trends with potential risk factors incorporated in further analysis.

## Electronic supplementary material

Supplementary file
