# Supplementary material for: The global epidemiology of bladder cancer: a joinpoint regression analysis of its incidence and mortality trends and projection
Source: Sci Rep. 2018 Jan 18;8:1129. doi: 10.1038/s41598-018-19199-z (PMC5773684; doi:10.1038/s41598-018-19199-z)
Supplement: Supplementary file 1 — Supplementary file [file 41598_2018_19199_MOESM1_ESM.pdf]

The global epidemiology of bladder cancer: a joinpoint regression analysis of its incidence and mortality trends and projection

Martin CS Wong MD, MPH, Franklin DH Fung BHSc (Hons), Colette Leung BSc (Hons), Wilson WL Cheung BSc (Hons), Bill W Goggins DSc (Harvard), CF Ng, MD

Supplementary Figure 1 The age-standardized incidence and mortality rates of bladder cancer in different countries (2012)

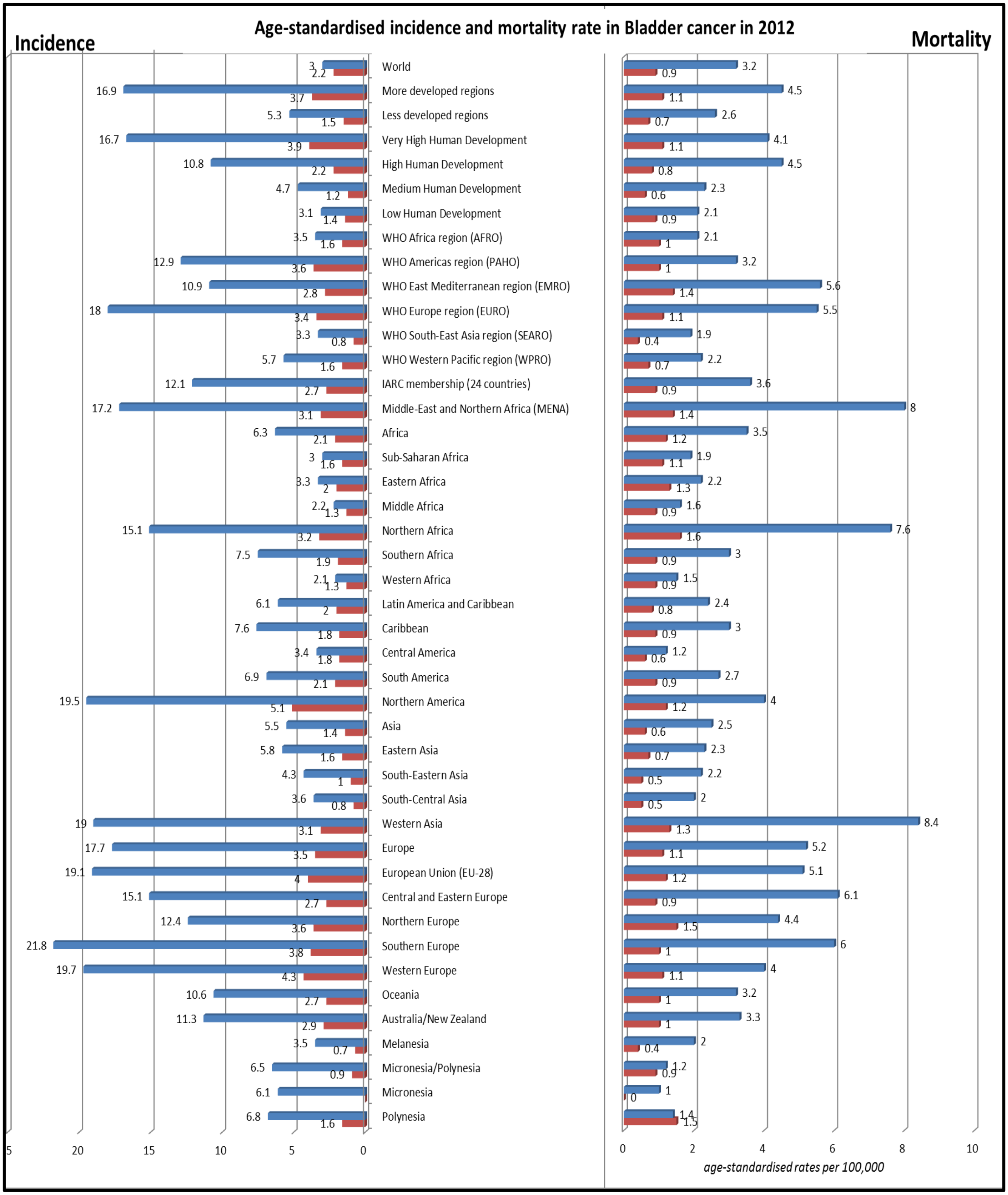

Blue: Male; Red: Female

Supplementary Figure 2 Findings from the joinpoint regression analysis of the global incidence rates of bladder cancer (Left: Male, Right: Female)

1) Latin America and the Caribbean

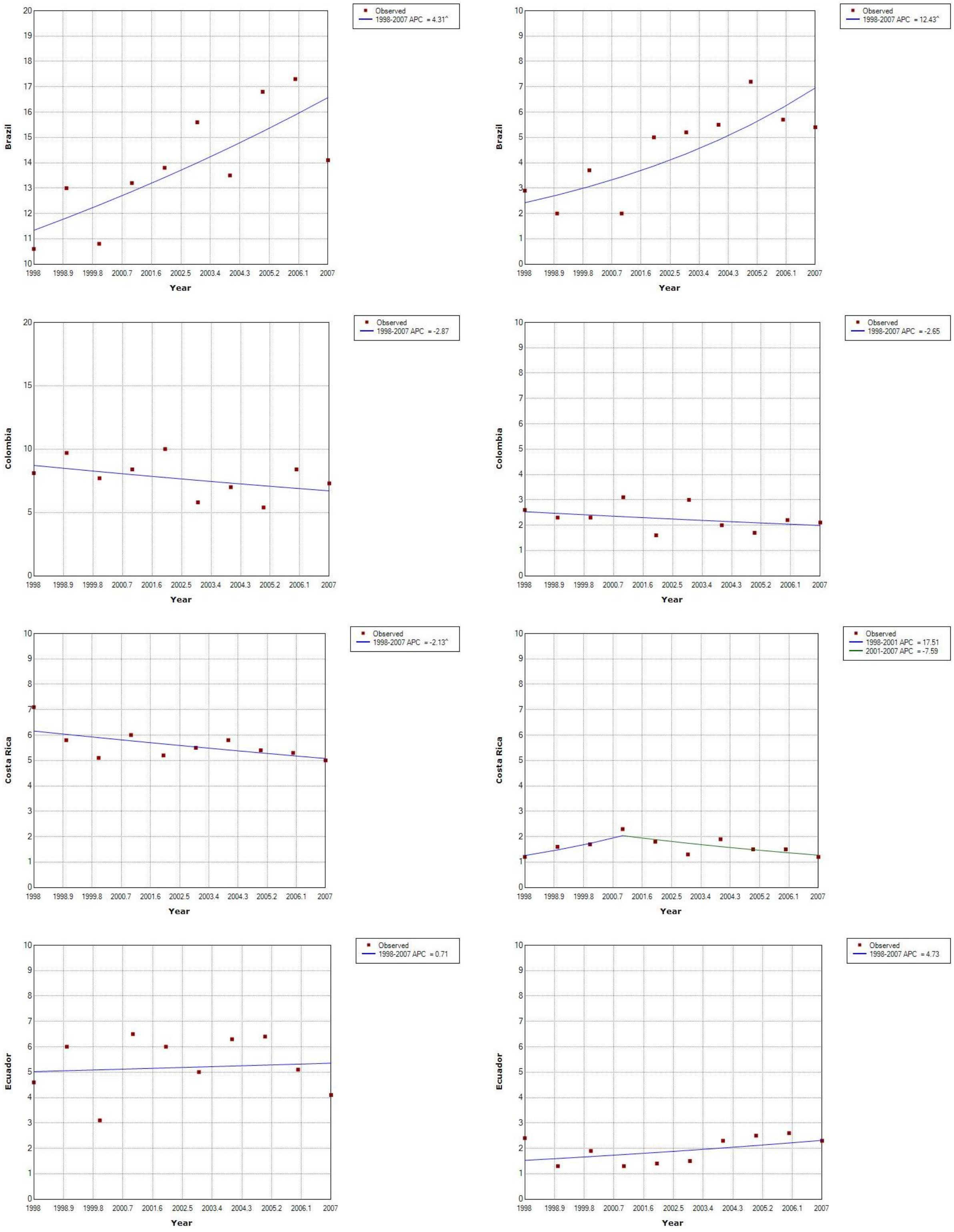

2) Northern America

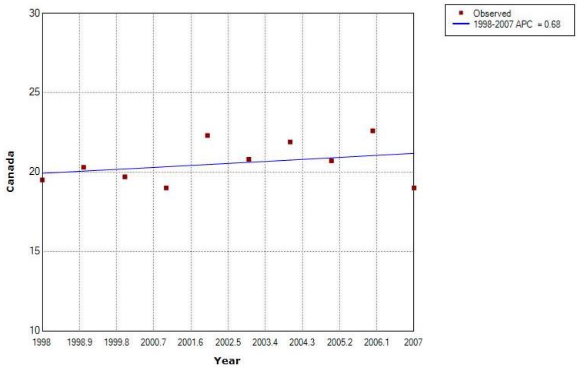

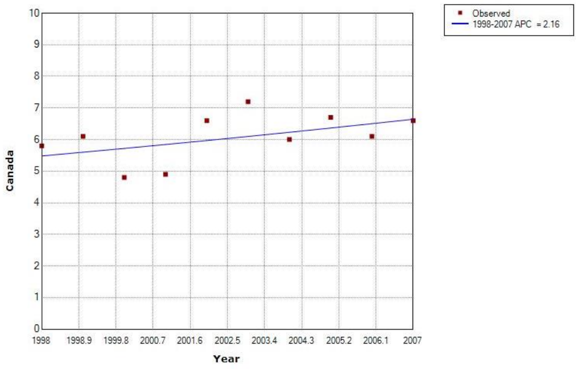

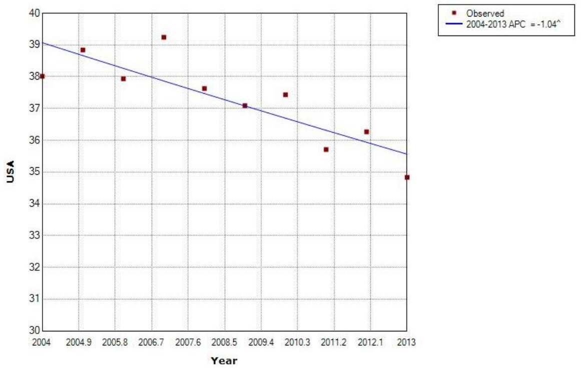

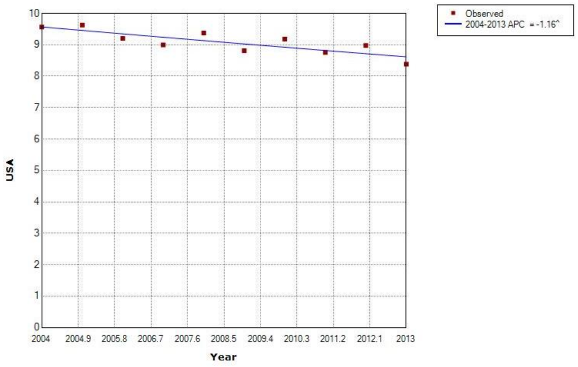

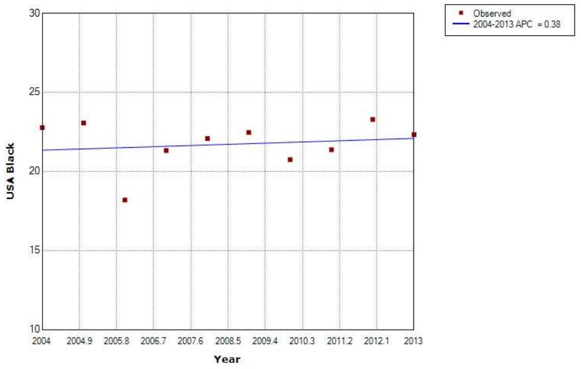

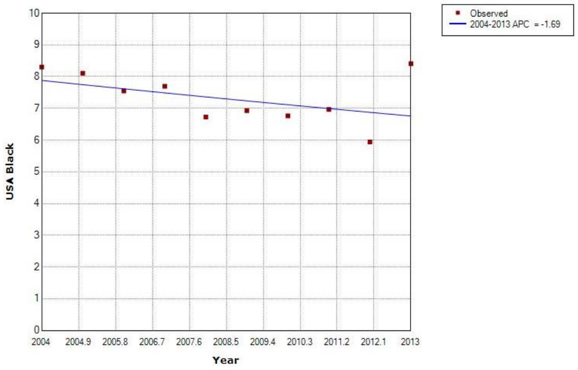

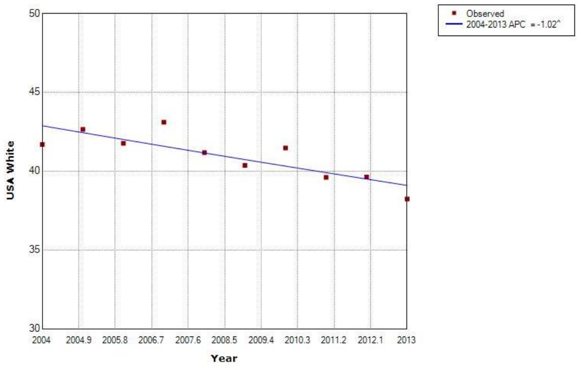

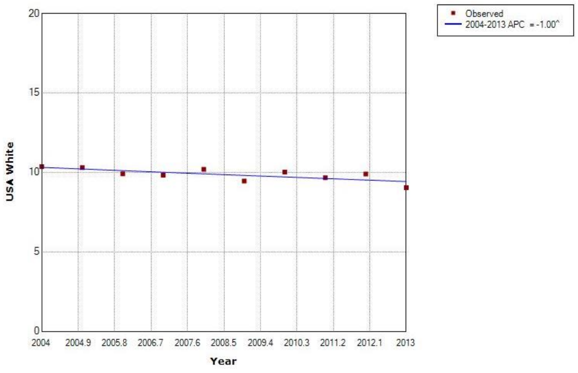

3) Asia

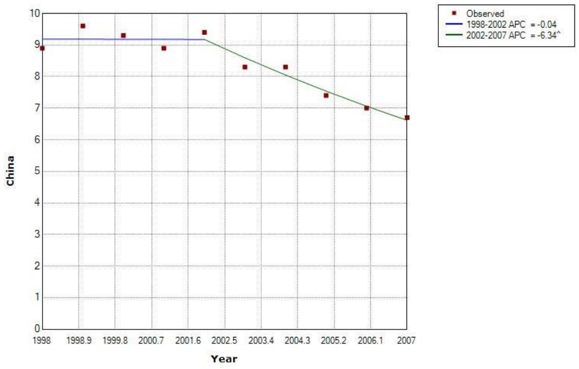
Observed
1998-2002 APC = -0.04
2002-2007 APC = -6.34^
China
Year

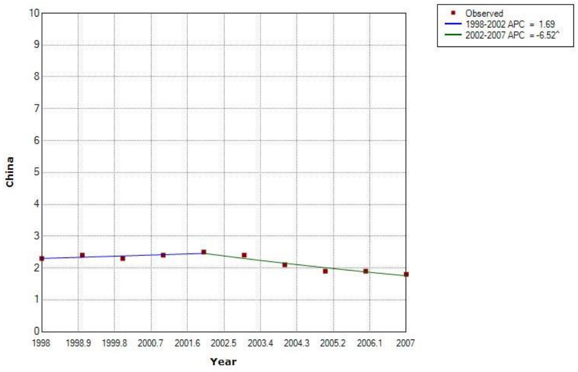
Observed
1998-2002 APC = 1.69
2002-2007 APC = -6.52^
China
Year

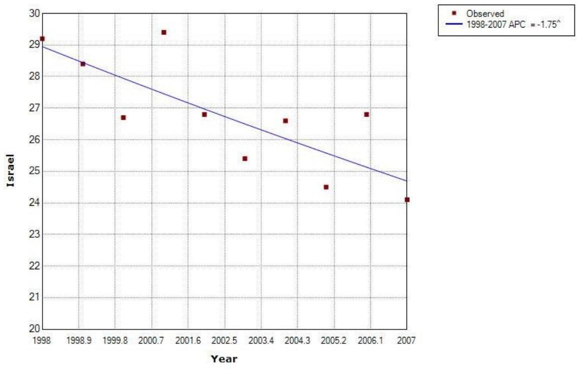
Observed
1998-2007 APC = -1.75^
Israel
Year

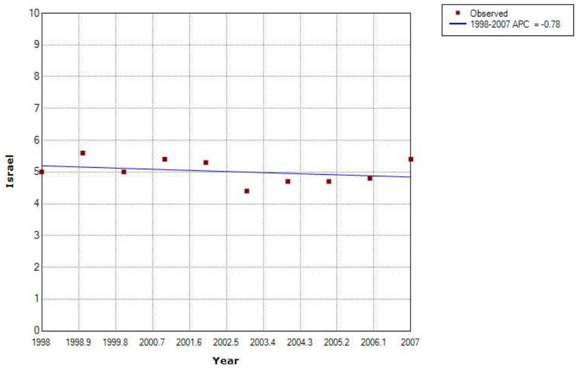
Observed
1998-2007 APC = -0.78
Israel
Year

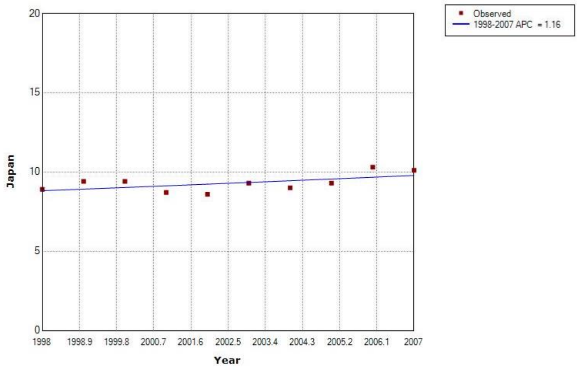
Observed
1998-2007 APC = 1.16
Japan
Year

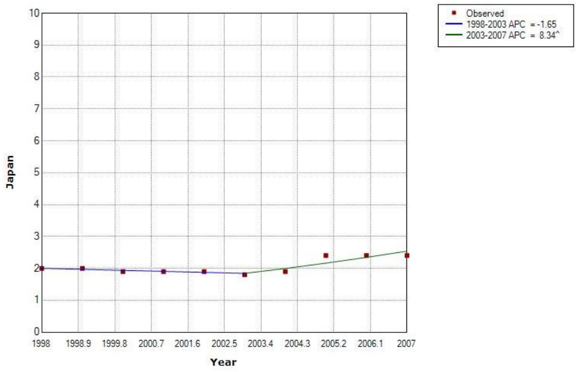
Observed
1998-2003 APC = -1.65
2003-2007 APC = 8.34^
Japan
Year

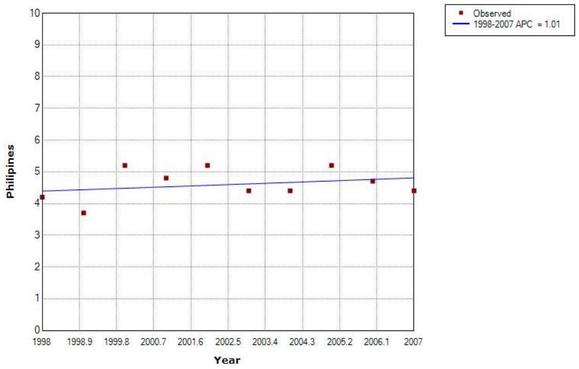
Observed
1998-2007 APC = 1.01
Philipines
Year

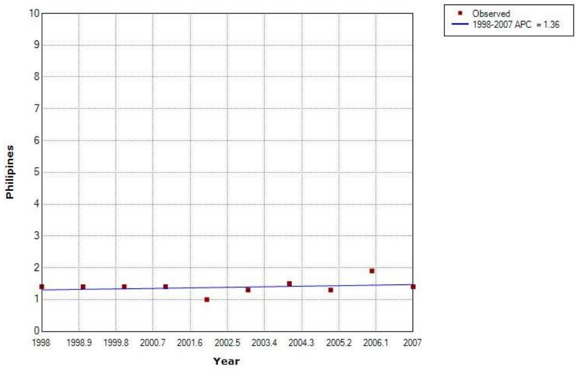
Observed
1998-2007 APC = 1.36
Philipines
Year

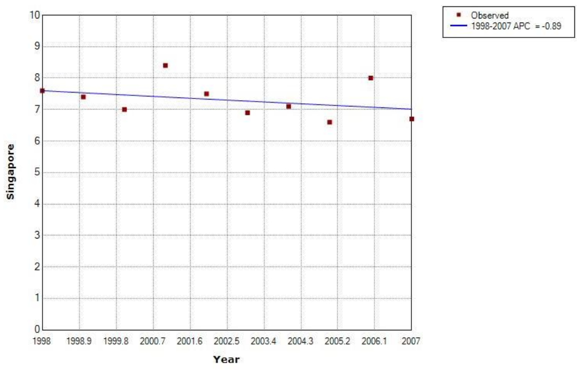
Observed
1998-2007 APC = -0.89
Singapore
Year

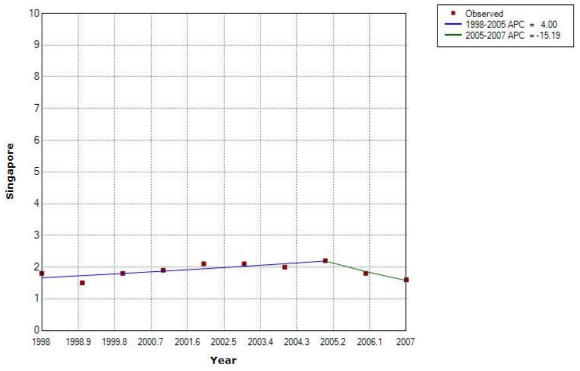
Observed
1998-2005 APC = 4.00
2005-2007 APC = -15.19
Singapore
Year

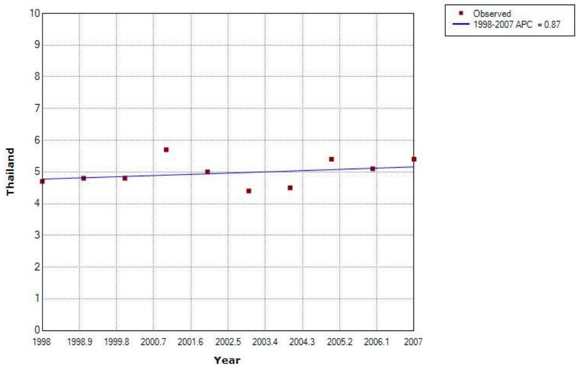
Observed
1998-2007 APC = 0.87
Thailand
Year

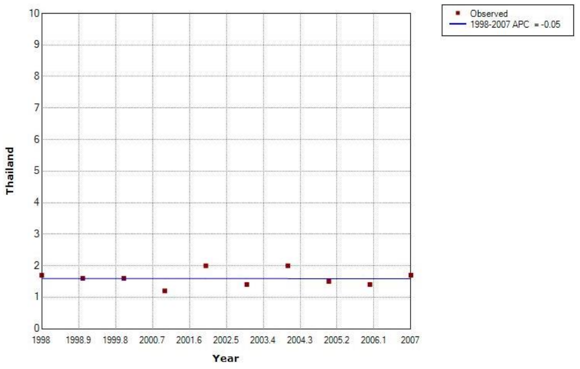
Observed
1998-2007 APC = -0.05
Thailand
Year

4) Oceania

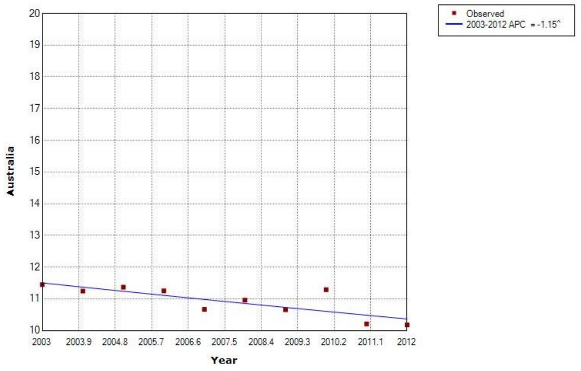
Observed
2003-2012 APC = -1.15^
Australia
Year

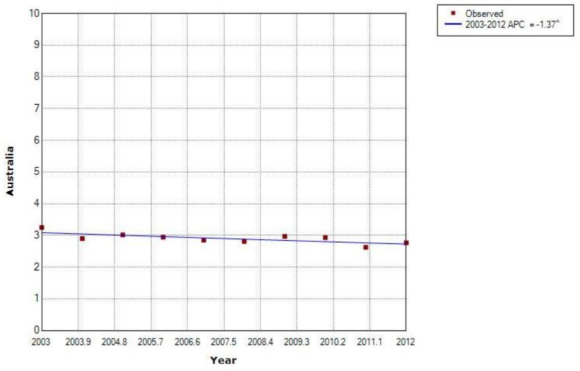
Observed
2003-2012 APC = -1.37^
Australia
Year

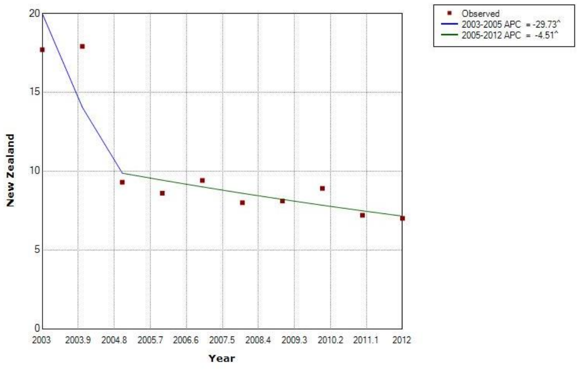
Observed
2003-2005 APC = -29.73^
2005-2012 APC = -4.51^
New Zealand
Year

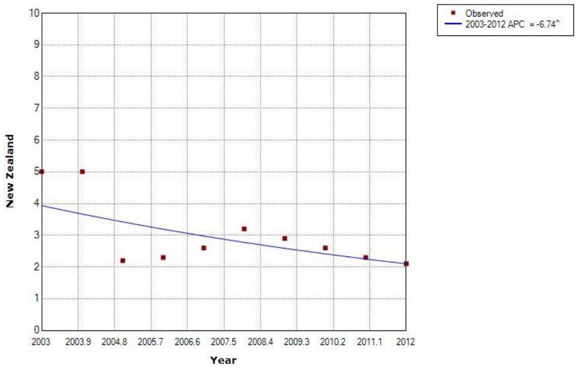
Observed
2003-2012 APC = -6.74^
New Zealand
Year

5) Northern Europe

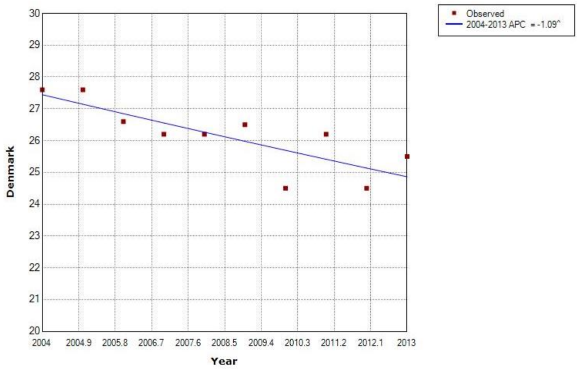

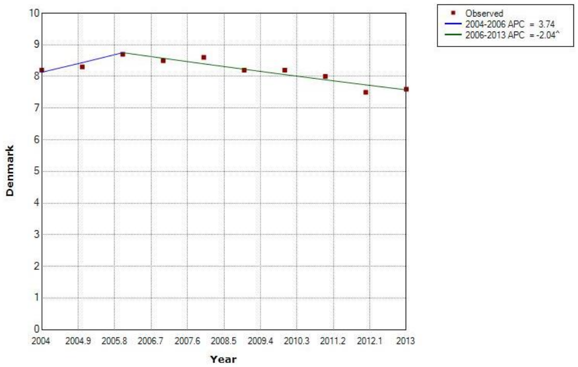

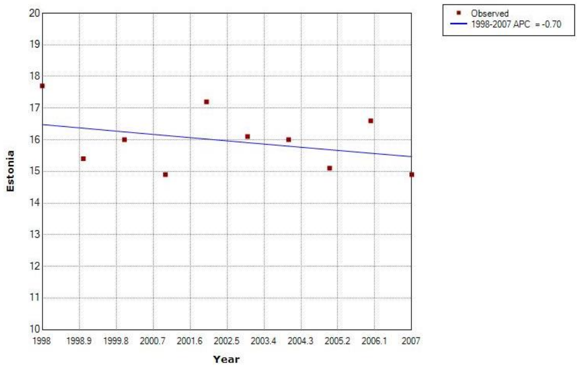

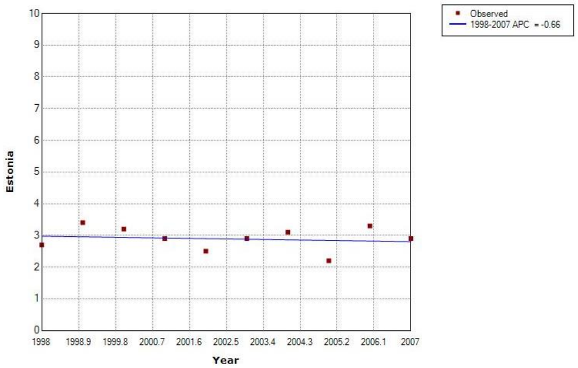

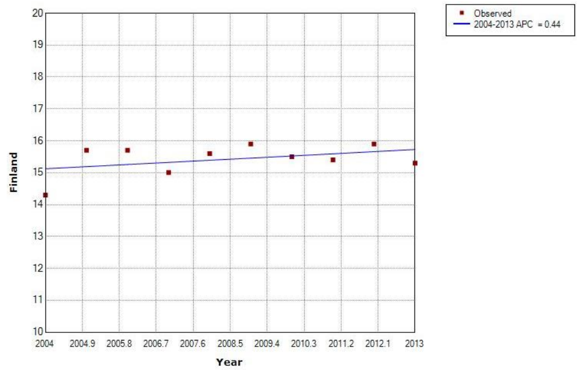

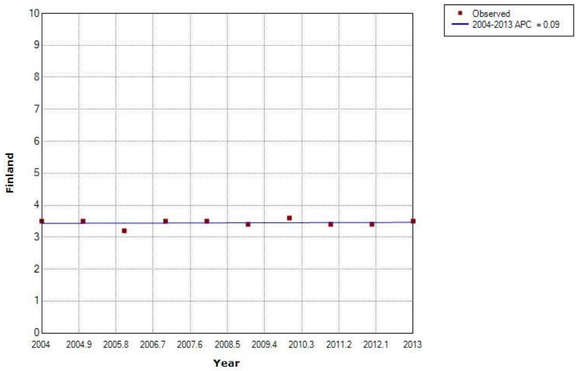

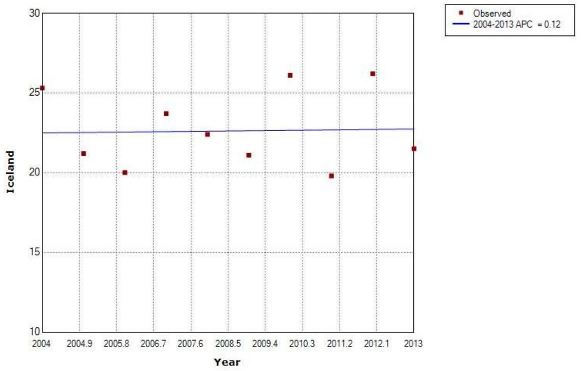

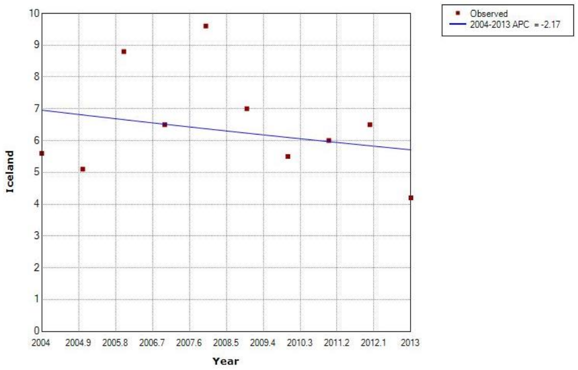

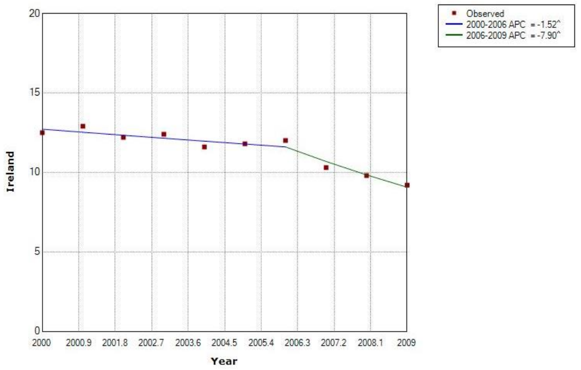
Observed
2000-2006 APC = -1.52^
2006-2009 APC = -7.90^
Ireland
Year

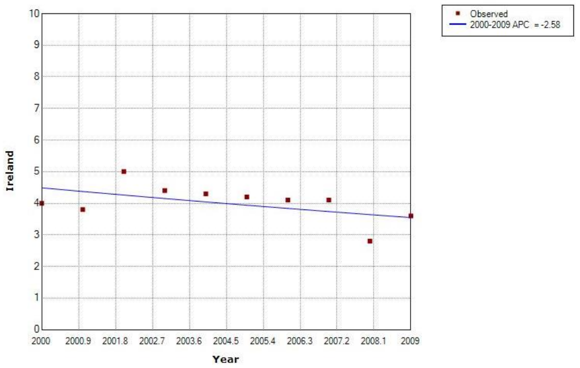
Observed
2000-2009 APC = -2.58
Ireland
Year

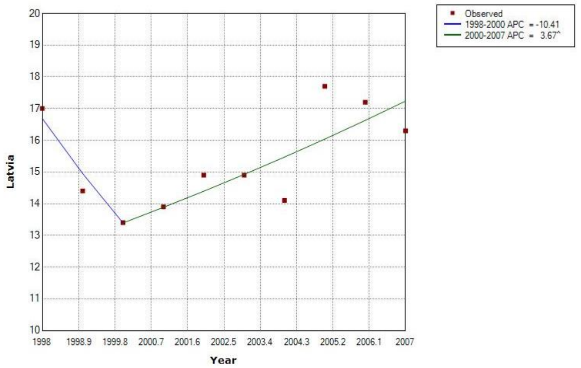
Observed
1998-2000 APC = -10.41
2000-2007 APC = 3.67^
Latvia
Year

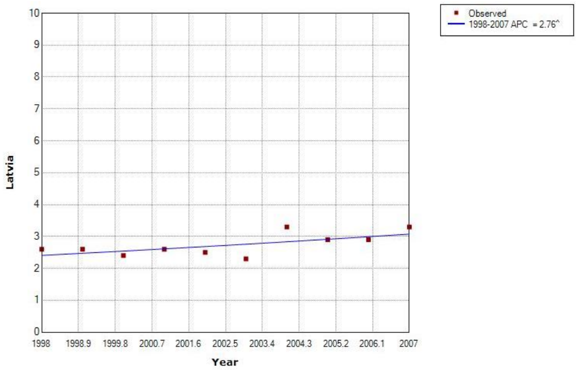
Observed
1998-2007 APC = 2.76^
Latvia
Year

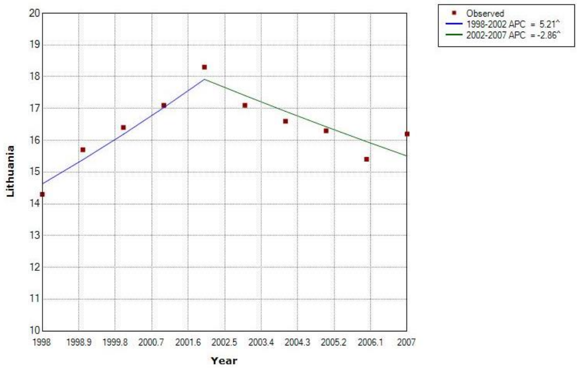
Observed
1998-2002 APC = 5.21^
2002-2007 APC = -2.86^
Lithuania
Year

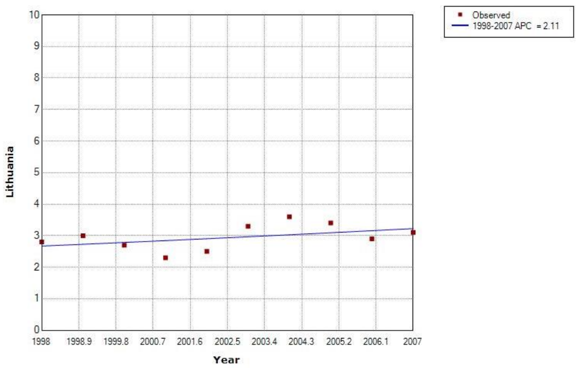
Observed
1998-2007 APC = 2.11
Lithuania
Year

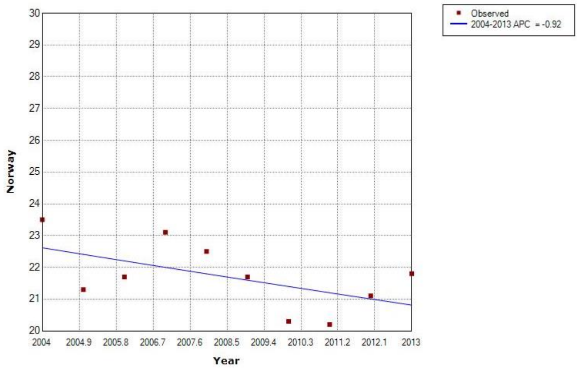
Observed
2004-2013 APC = -0.92
Norway
Year

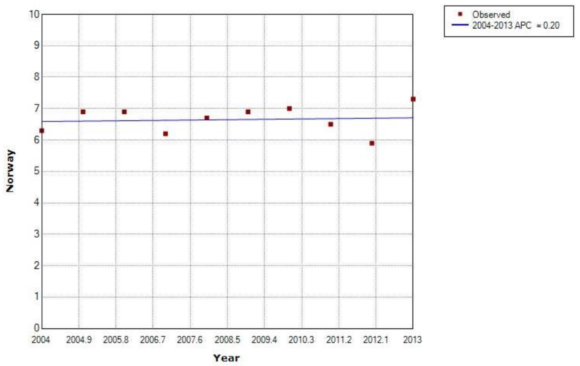
Observed
2004-2013 APC = 0.20
Norway
Year

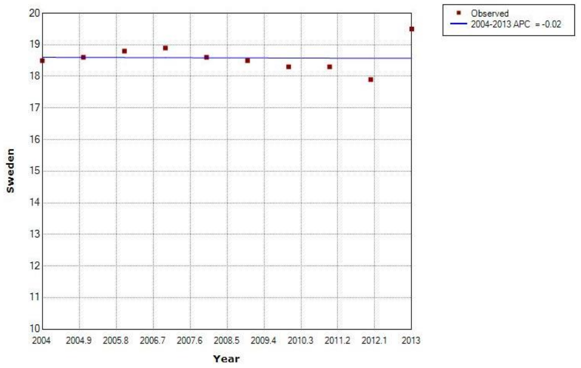
Observed
2004-2013 APC = -0.02
Sweden
Year

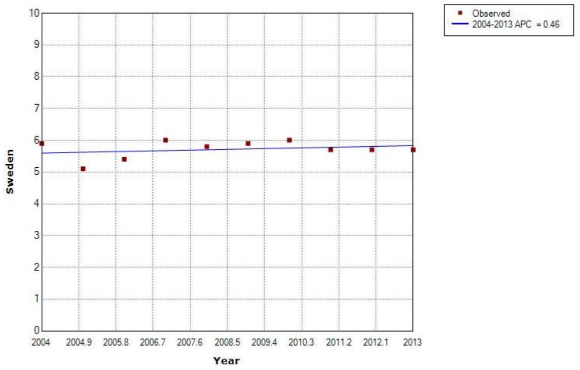
Observed
2004-2013 APC = 0.46
Sweden
Year

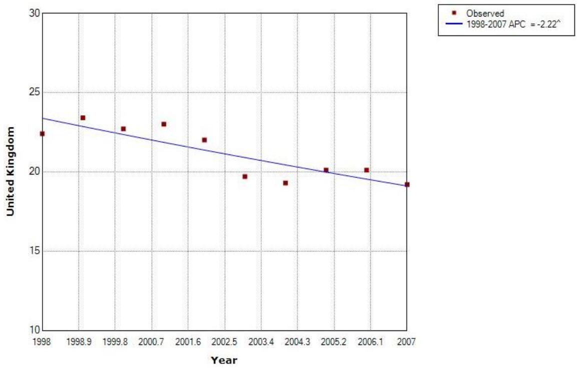

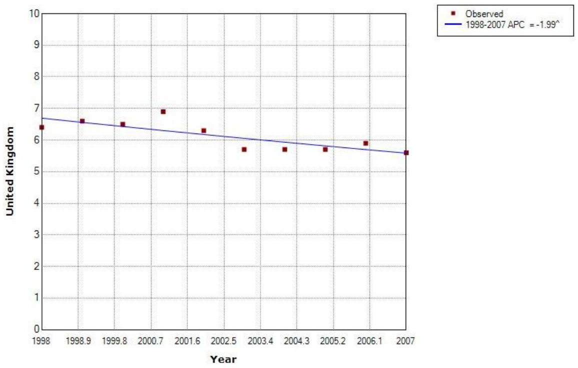

6) Western Europe

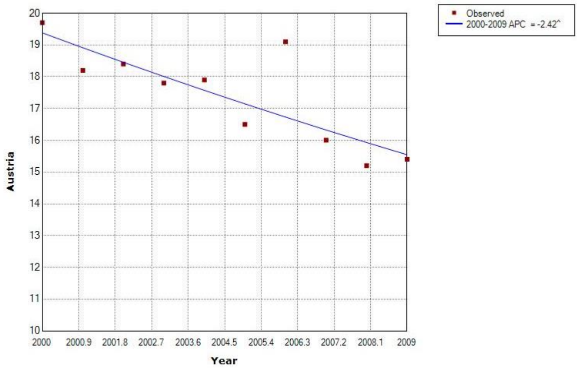

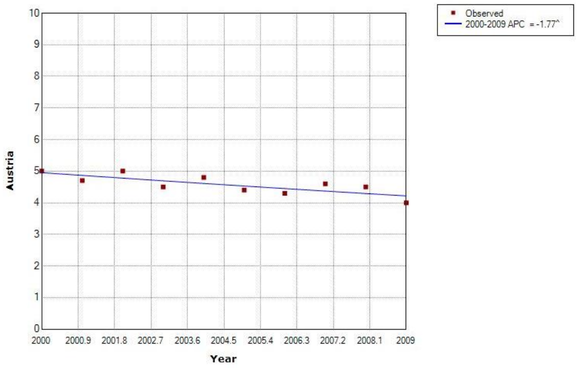

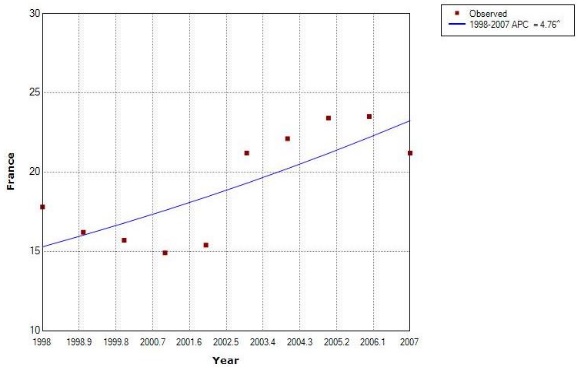

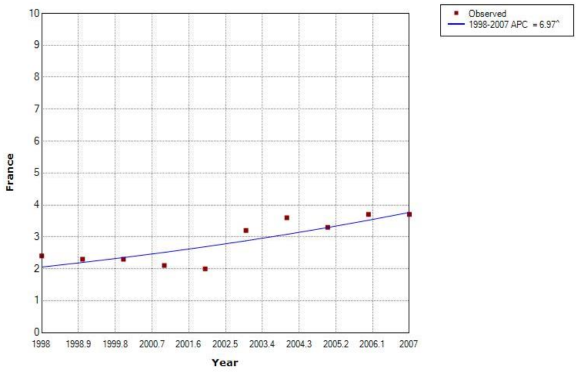

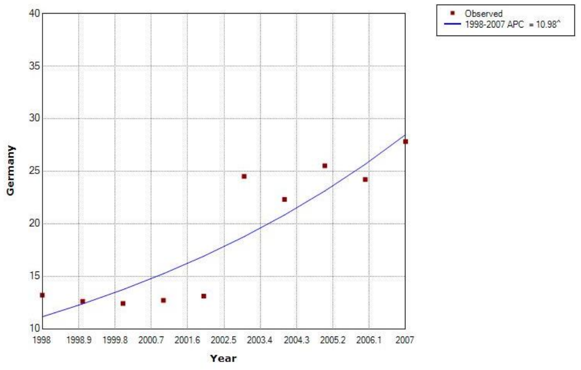

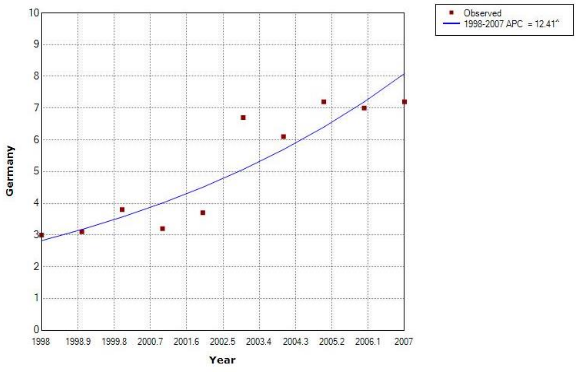

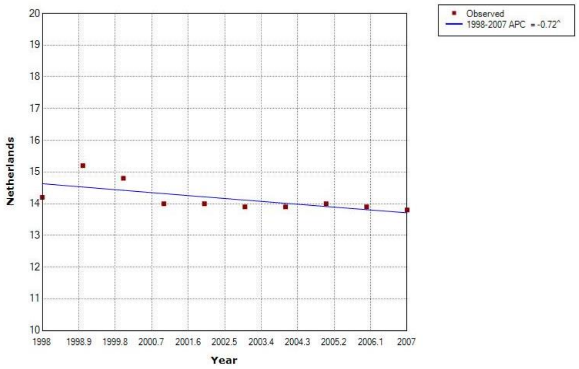

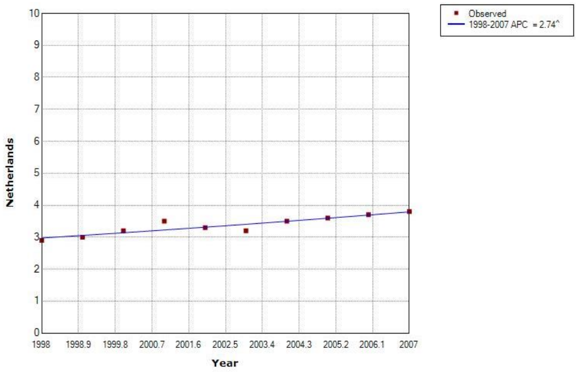

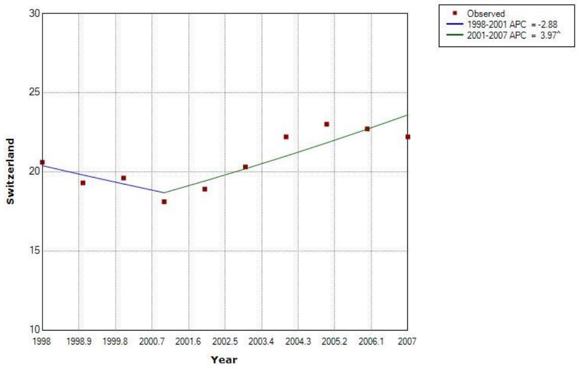

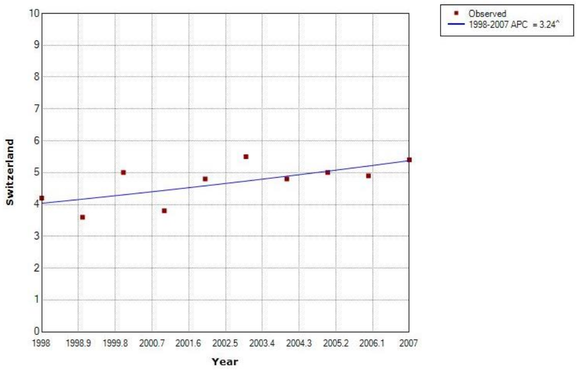

7) Southern Europe

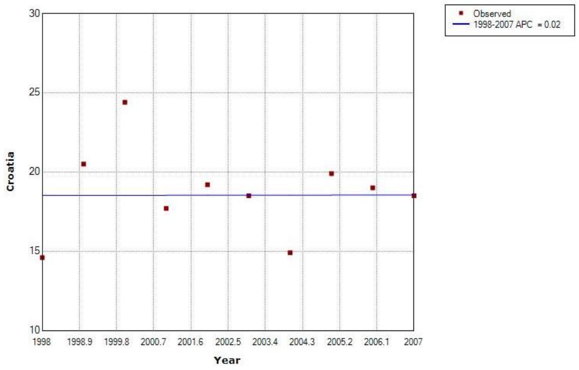

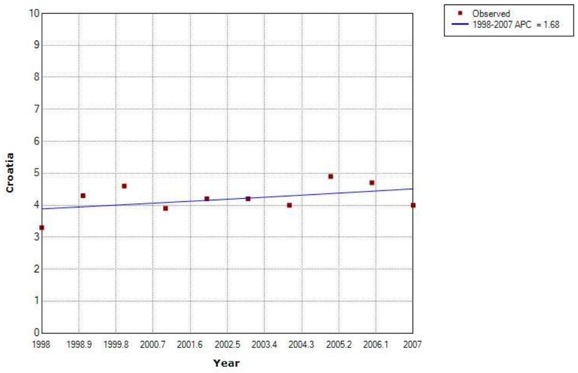

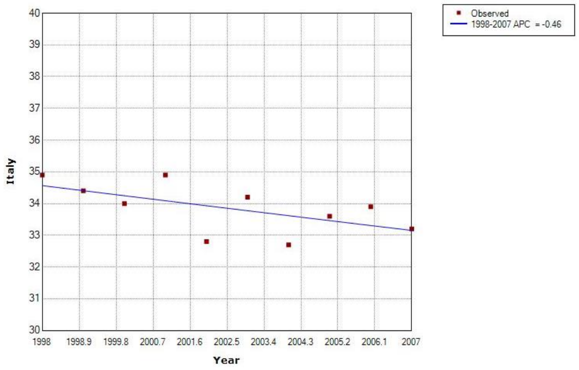

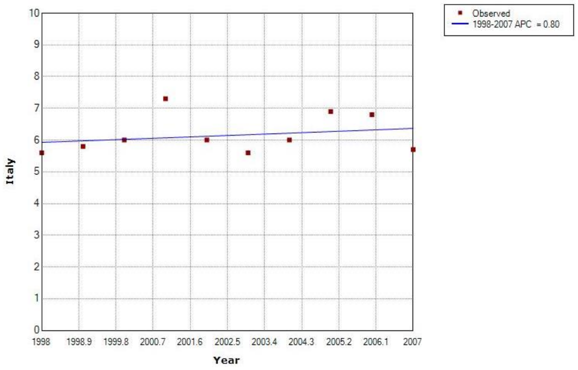

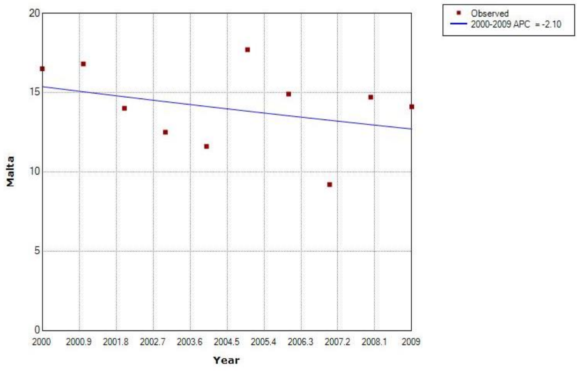

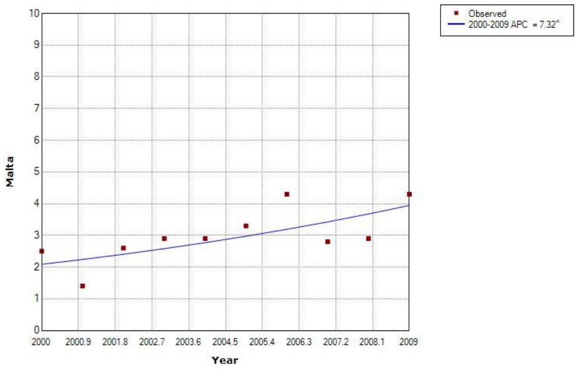

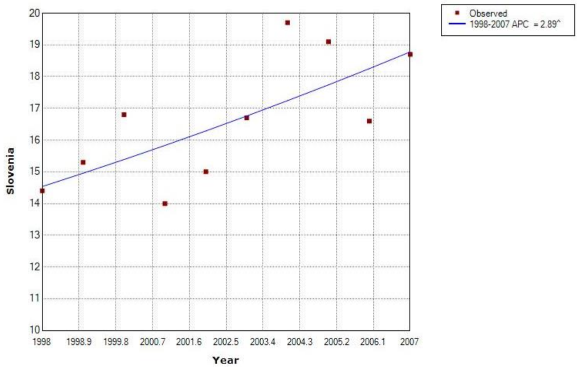

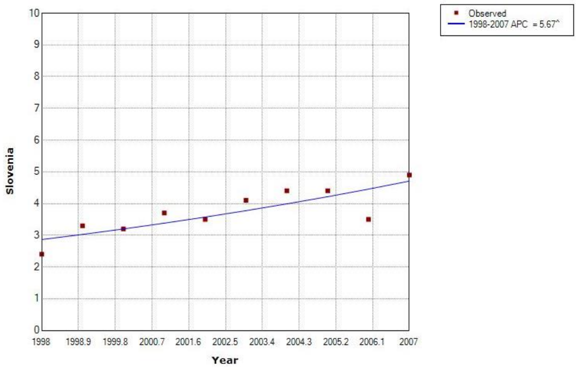

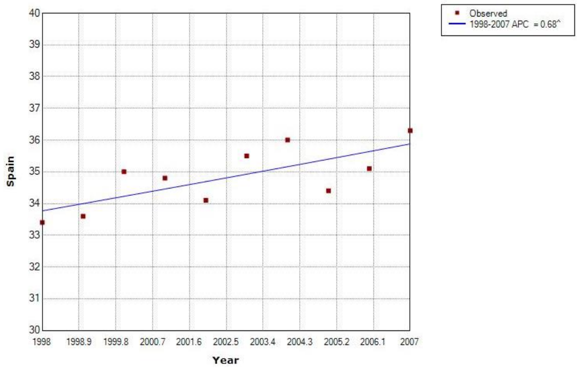

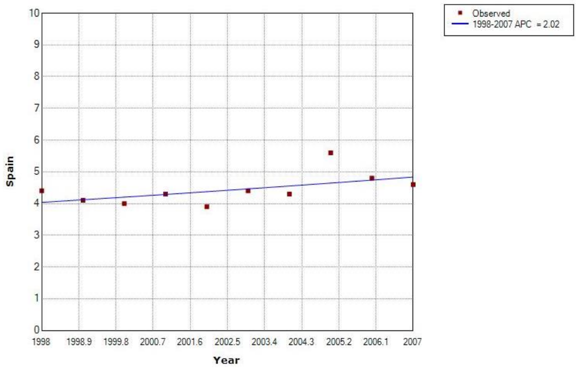

8) Eastern Europe

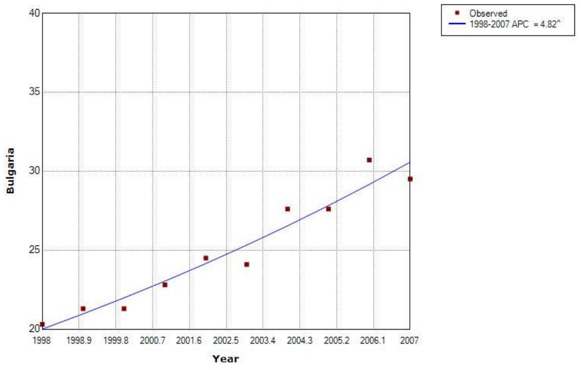

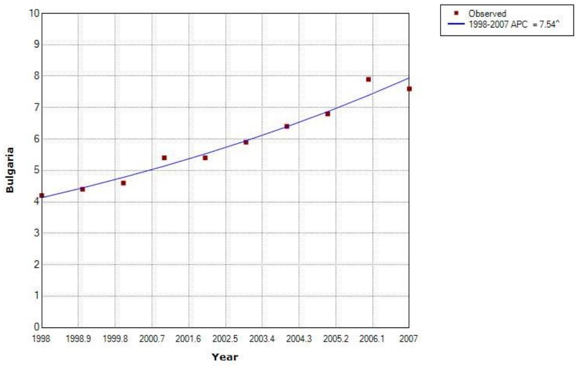

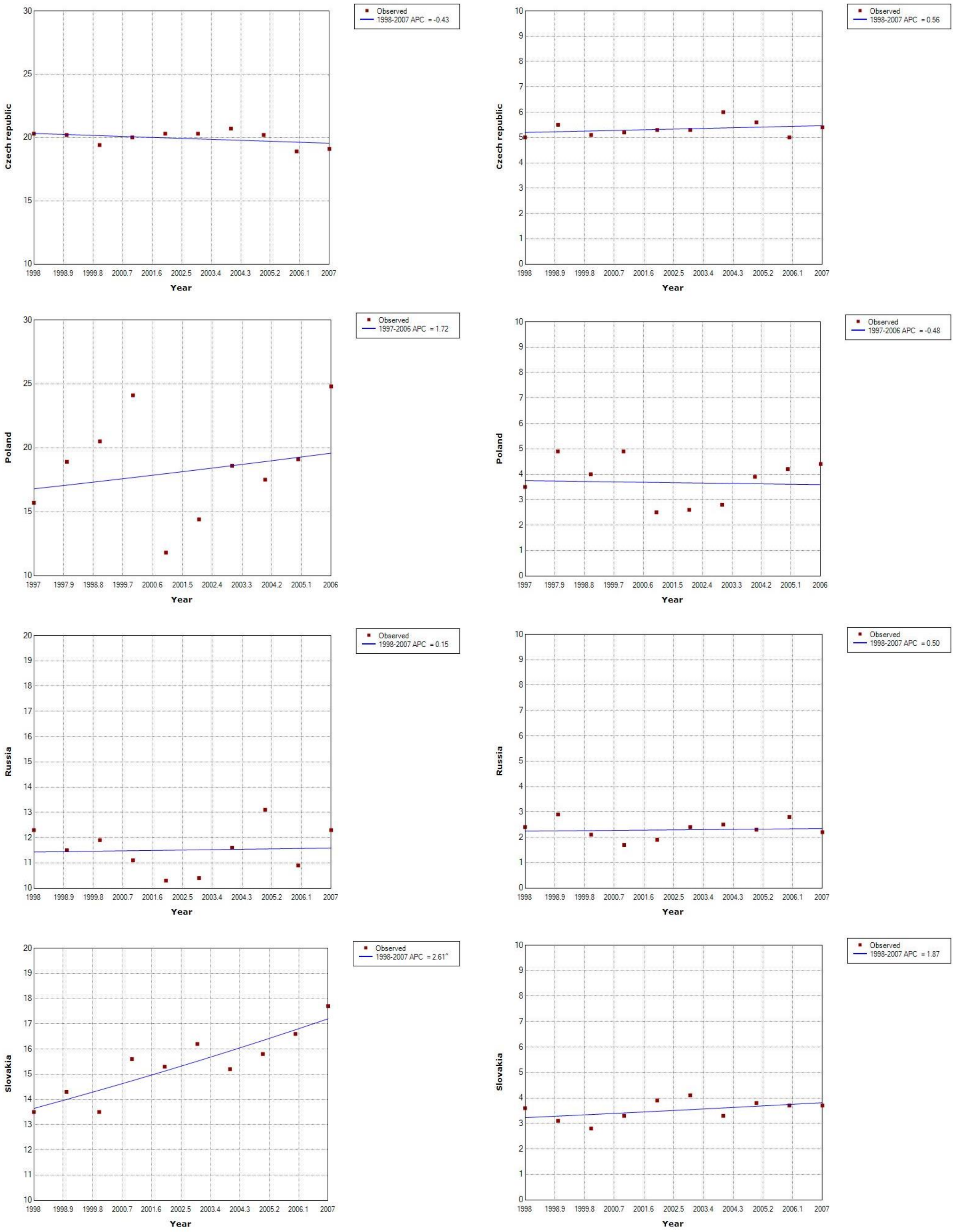

Czech republic
Year
Observed
1998-2007 APC = -0.43
1998-2007 APC = 0.56
Poland
1997-2006 APC = 1.72
1997-2006 APC = -0.48
Russia
1998-2007 APC = 0.15
1998-2007 APC = 0.50
Slovakia
1998-2007 APC = 2.61^
1998-2007 APC = 1.87

Supplementary Figure 3 Findings from the joinpoint regression analysis of the global mortality rates of bladder cancer (Left: Male, Right: Female)

1) Latin America and the Caribbean

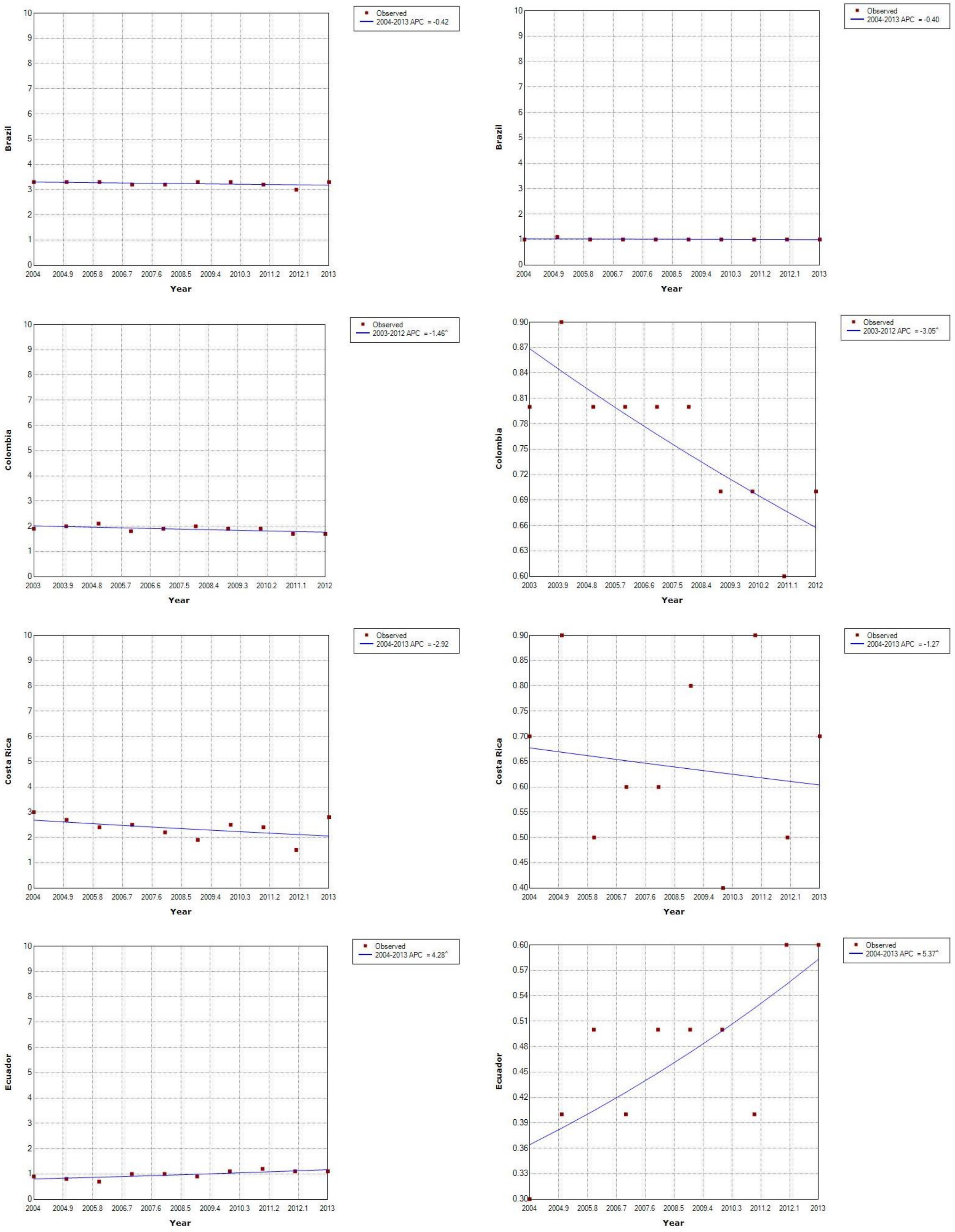

2) Northern America

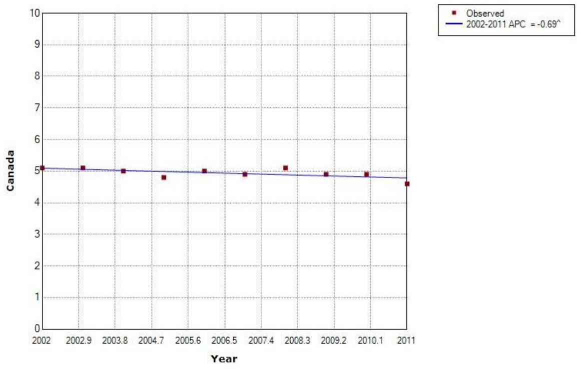

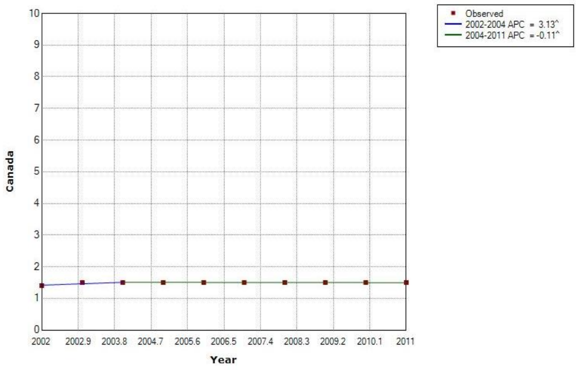

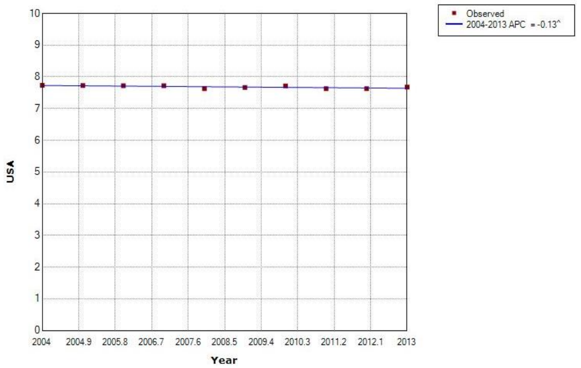

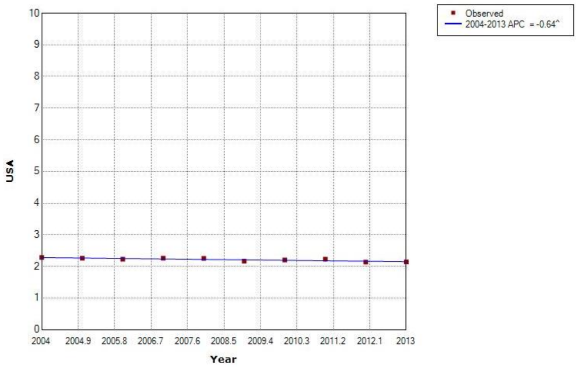

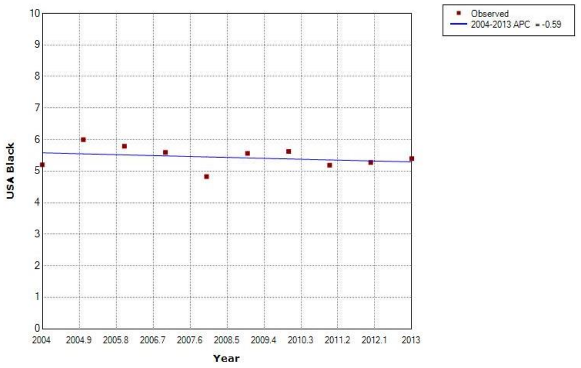

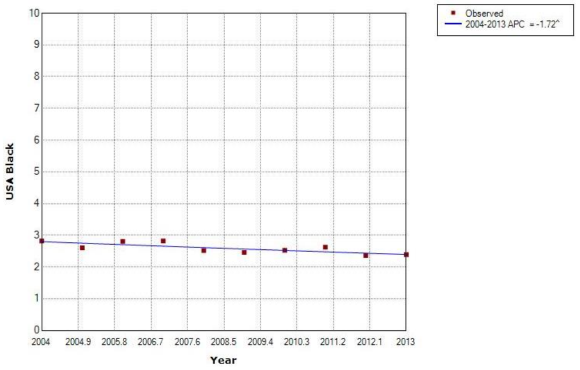

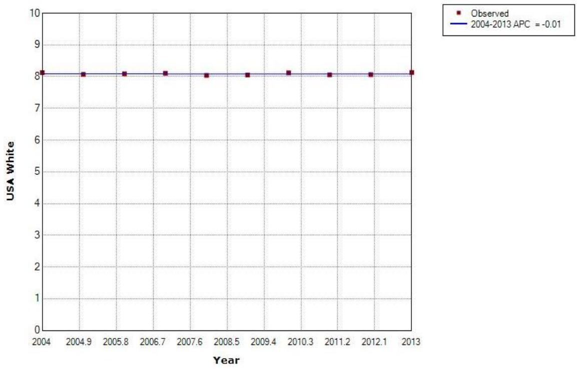

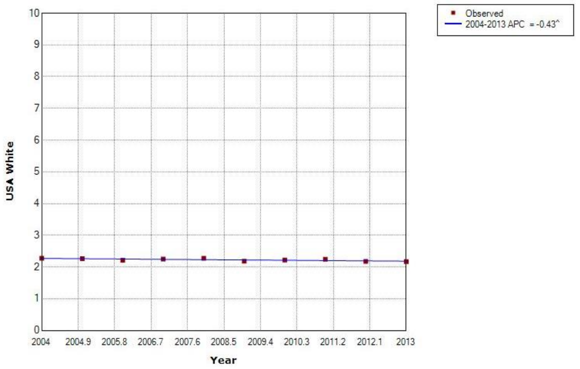

3) Asia

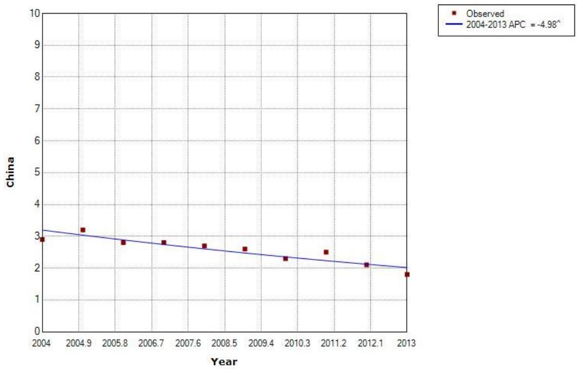
Observed
2004-2013 APC = -4.98^
China
Year

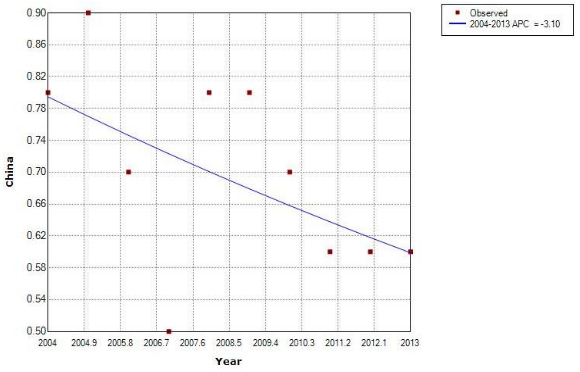
Observed
2004-2013 APC = -3.10
China
Year

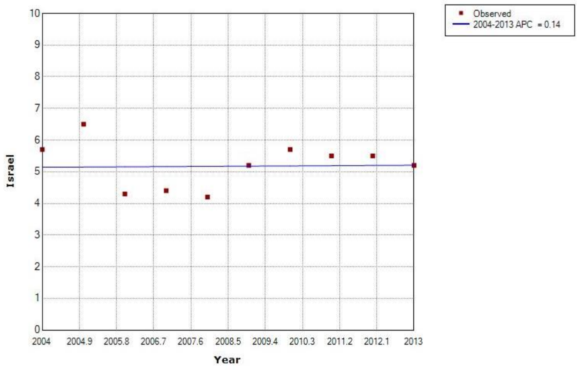
Observed
2004-2013 APC = 0.14
Israel
Year

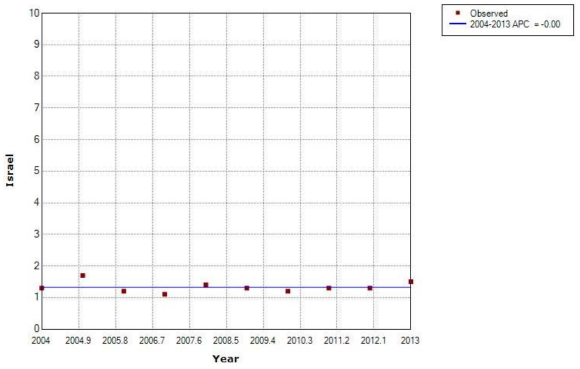
Observed
2004-2013 APC = -0.00
Israel
Year

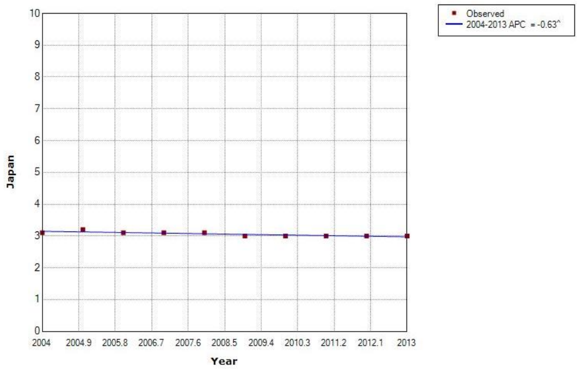
Observed
2004-2013 APC = -0.63^
Japan
Year

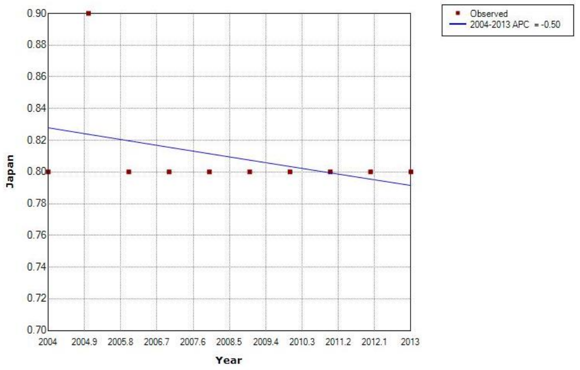
Observed
2004-2013 APC = -0.50
Japan
Year

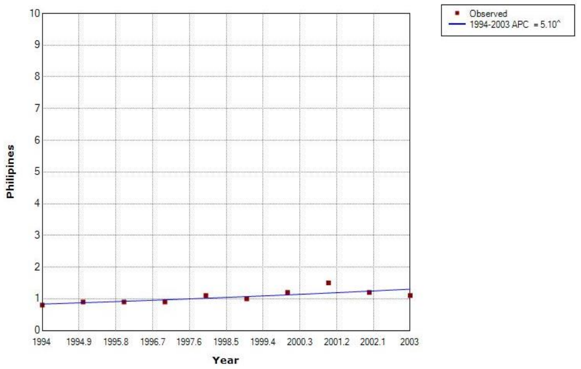
Observed
1994-2003 APC = 5.10^
Philipines
Year

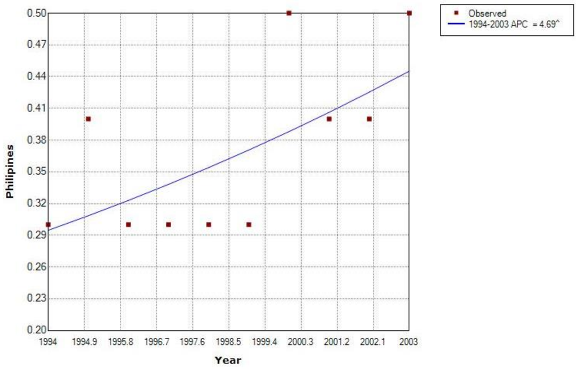
Observed
1994-2003 APC = 4.69^
Philipines
Year

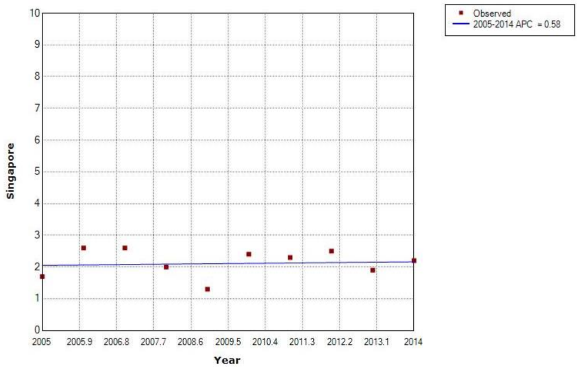

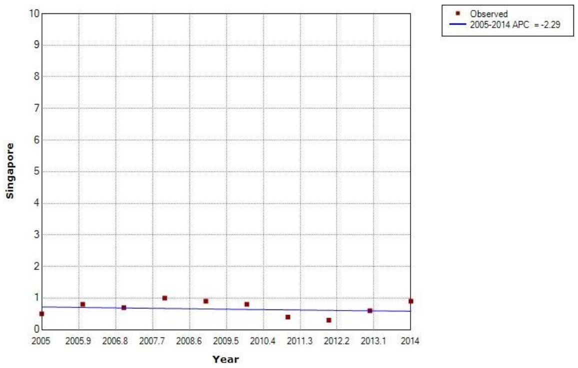

4) Oceania

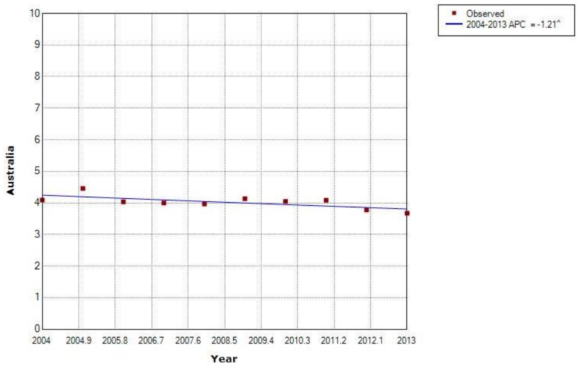

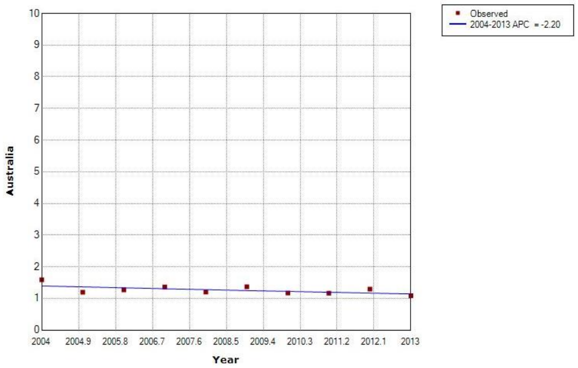

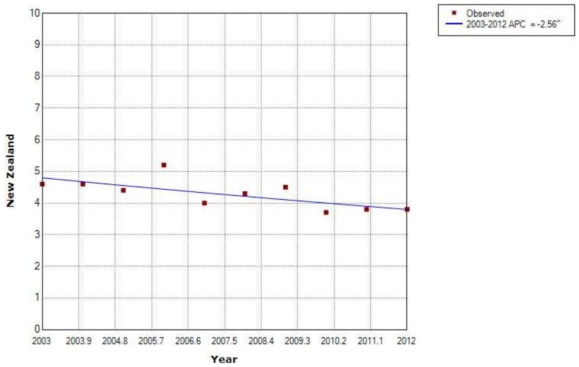

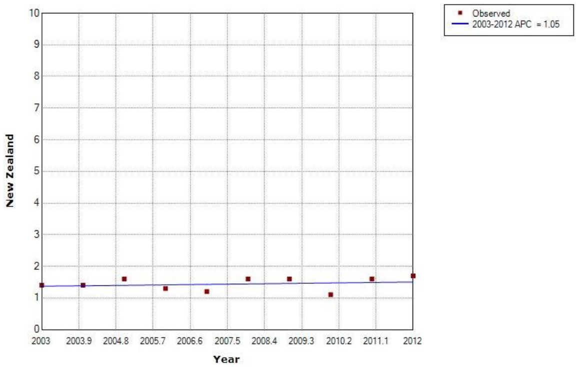

5) Northern Europe

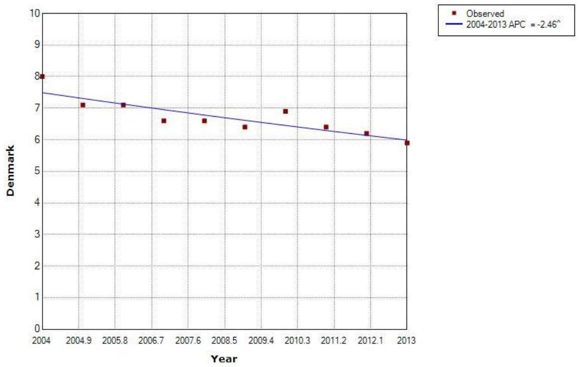

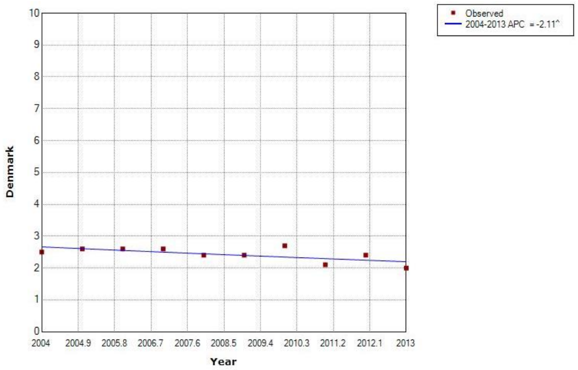

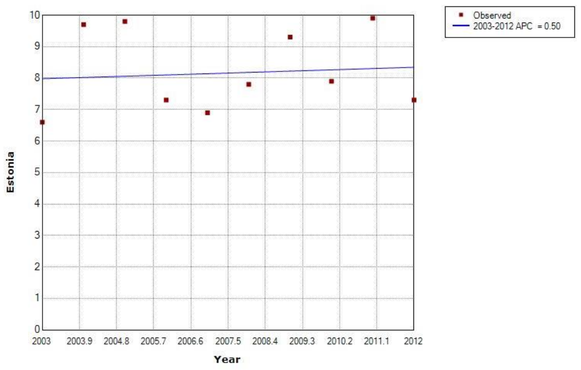
Observed
2003-2012 APC = 0.50
Estonia
Year
0 1 2 3 4 5 6 7 8 9 10
2003 2003.9 2004.8 2005.7 2006.6 2007.5 2008.4 2009.3 2010.2 2011.1 2012

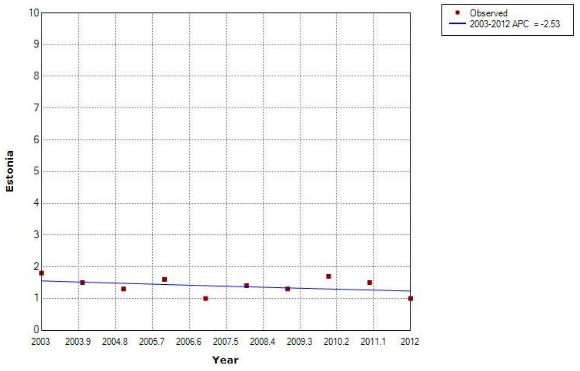
Observed
2003-2012 APC = -2.53
Estonia
Year
0 1 2 3 4 5 6 7 8 9 10
2003 2003.9 2004.8 2005.7 2006.6 2007.5 2008.4 2009.3 2010.2 2011.1 2012

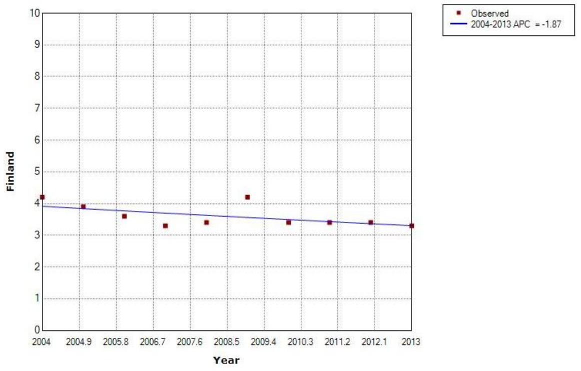
Observed
2004-2013 APC = -1.87
Finland
Year
0 1 2 3 4 5 6 7 8 9 10
2004 2004.9 2005.8 2006.7 2007.6 2008.5 2009.4 2010.3 2011.2 2012.1 2013

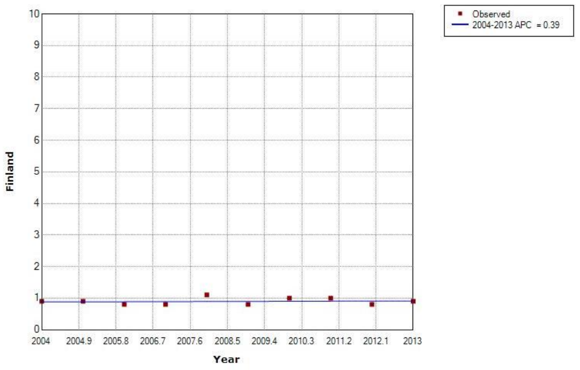
Observed
2004-2013 APC = 0.39
Finland
Year
0 1 2 3 4 5 6 7 8 9 10
2004 2004.9 2005.8 2006.7 2007.6 2008.5 2009.4 2010.3 2011.2 2012.1 2013

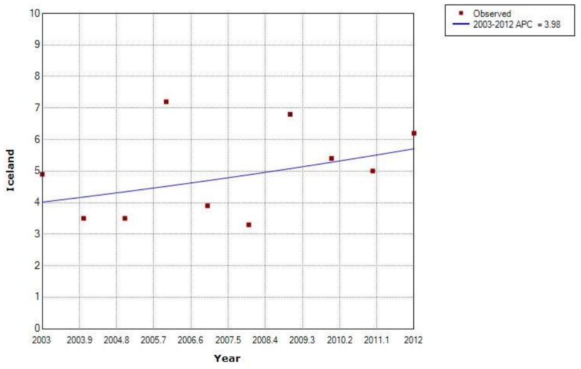
Observed
2003-2012 APC = 3.98
Iceland
Year
0 1 2 3 4 5 6 7 8 9 10
2003 2003.9 2004.8 2005.7 2006.6 2007.5 2008.4 2009.3 2010.2 2011.1 2012

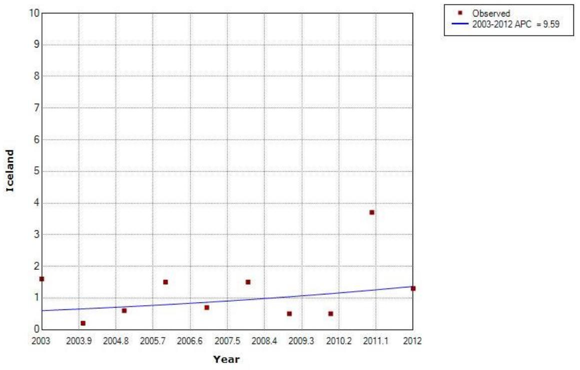
Observed
2003-2012 APC = 9.59
Iceland
Year
0 1 2 3 4 5 6 7 8 9 10
2003 2003.9 2004.8 2005.7 2006.6 2007.5 2008.4 2009.3 2010.2 2011.1 2012

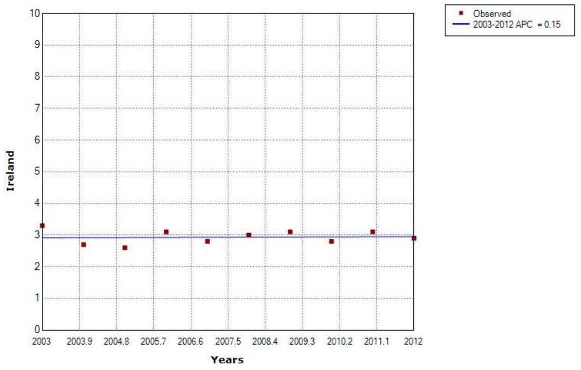
Observed
2003-2012 APC = 0.15
Ireland
Years
0 1 2 3 4 5 6 7 8 9 10
2003 2003.9 2004.8 2005.7 2006.6 2007.5 2008.4 2009.3 2010.2 2011.1 2012

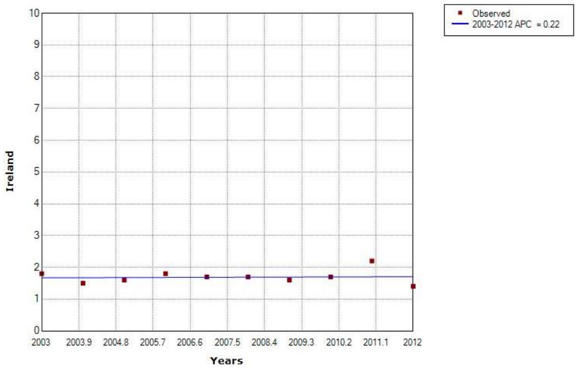
Observed
2003-2012 APC = 0.22
Ireland
Years
0 1 2 3 4 5 6 7 8 9 10
2003 2003.9 2004.8 2005.7 2006.6 2007.5 2008.4 2009.3 2010.2 2011.1 2012

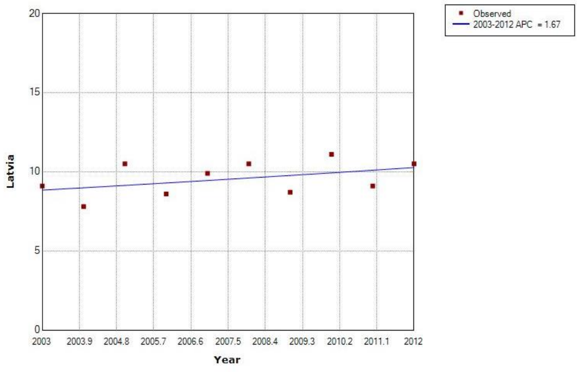
Observed
2003-2012 APC = 1.67
Latvia
Year
0 5 10 15 20
2003 2003.9 2004.8 2005.7 2006.6 2007.5 2008.4 2009.3 2010.2 2011.1 2012

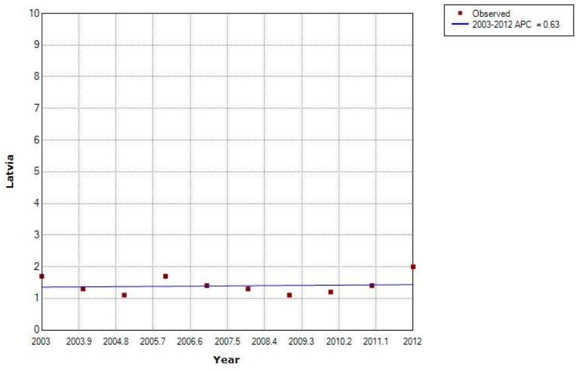
Observed
2003-2012 APC = 0.63
Latvia
Year
0 1 2 3 4 5 6 7 8 9 10
2003 2003.9 2004.8 2005.7 2006.6 2007.5 2008.4 2009.3 2010.2 2011.1 2012

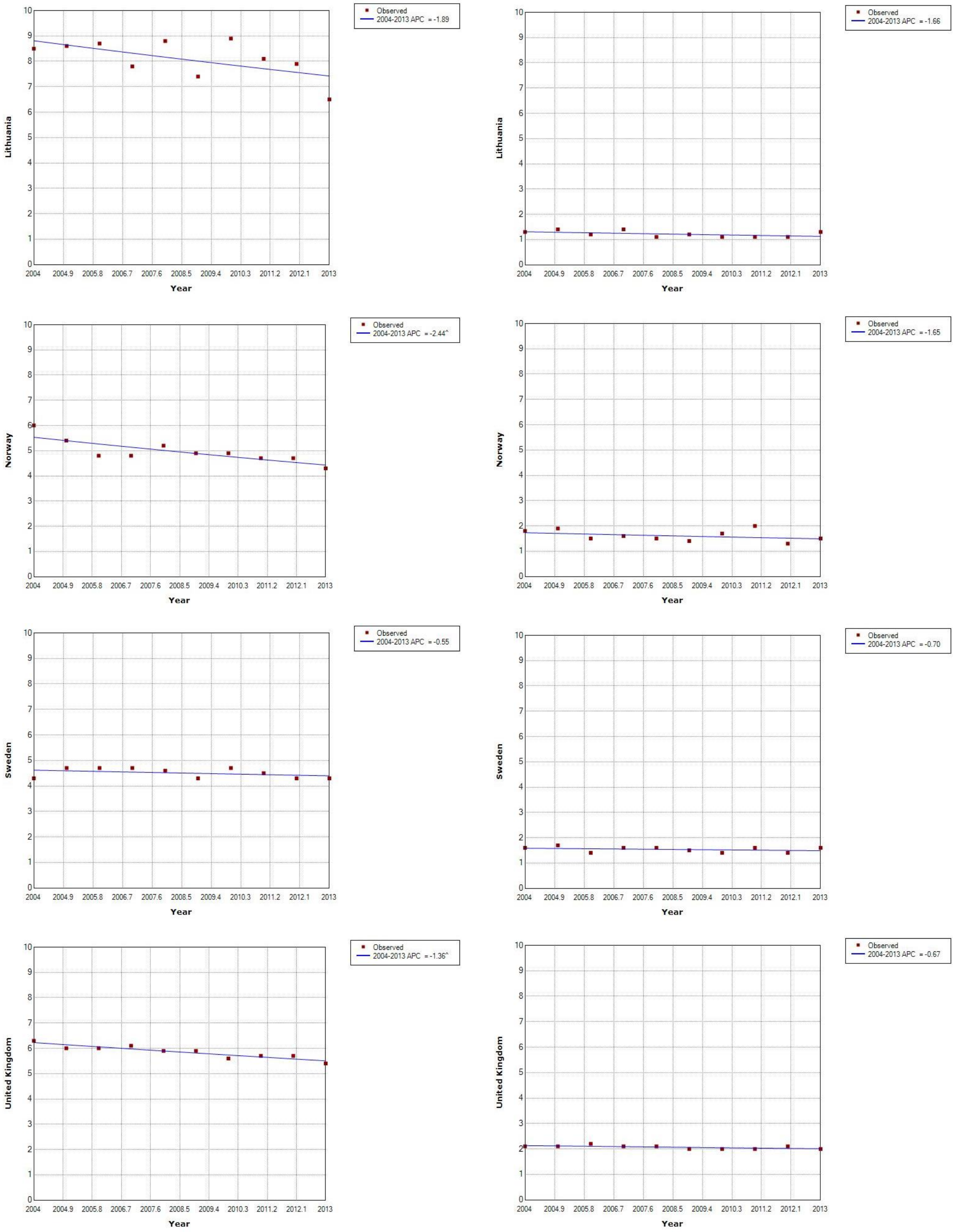
Lithuania
Observed
2004-2013 APC = -1.89
Lithuania
Observed
2004-2013 APC = -1.66
Norway
Observed
2004-2013 APC = -2.44^
Norway
Observed
2004-2013 APC = -1.65
Sweden
Observed
2004-2013 APC = -0.55
Sweden
Observed
2004-2013 APC = -0.70
United Kingdom
Observed
2004-2013 APC = -1.36^
United Kingdom
Observed
2004-2013 APC = -0.67
Year
2004
2004.9
2005.8
2006.7
2007.6
2008.5
2009.4
2010.3
2011.2
2012.1
2013

6) Western Europe

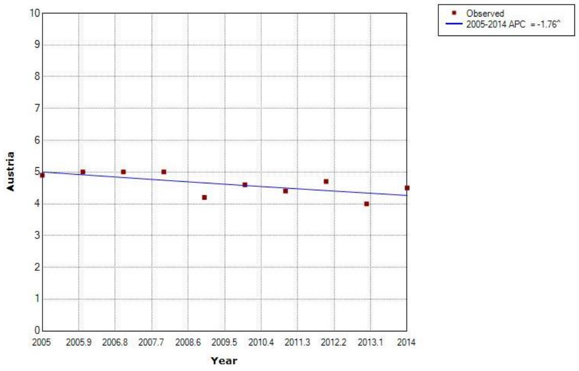

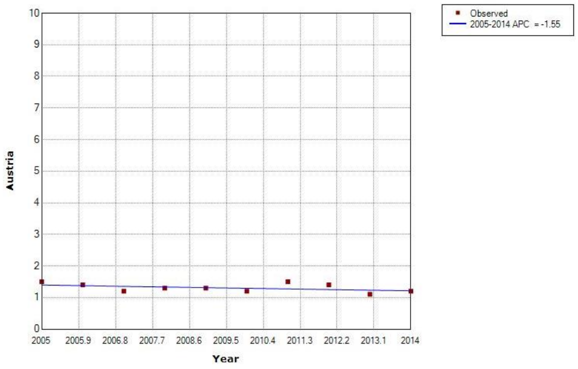

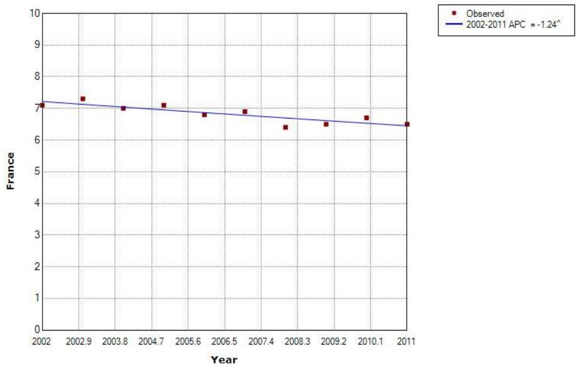

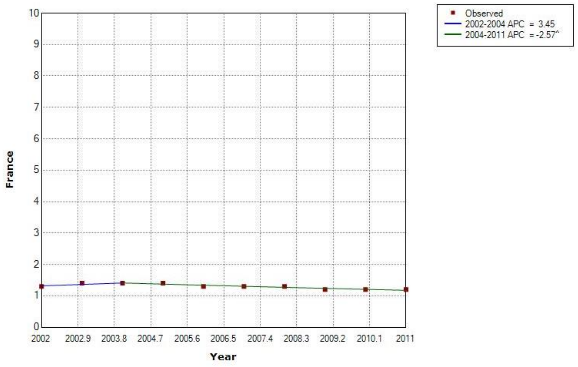

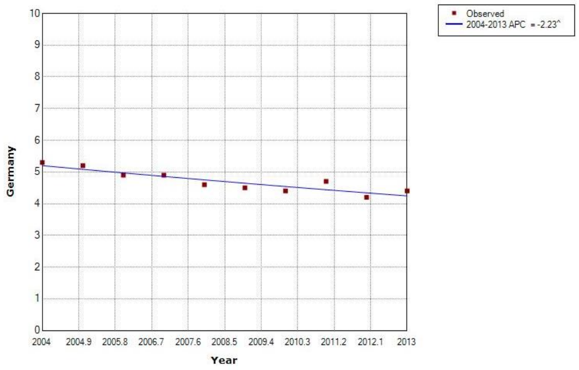

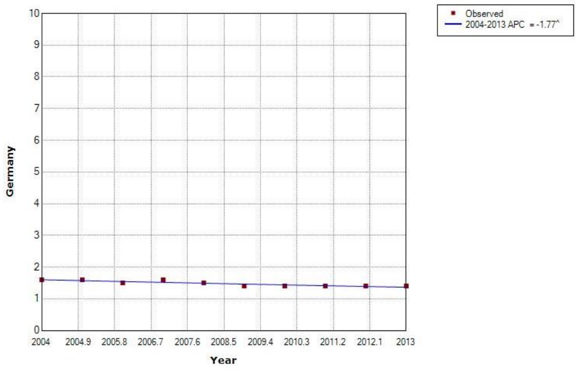

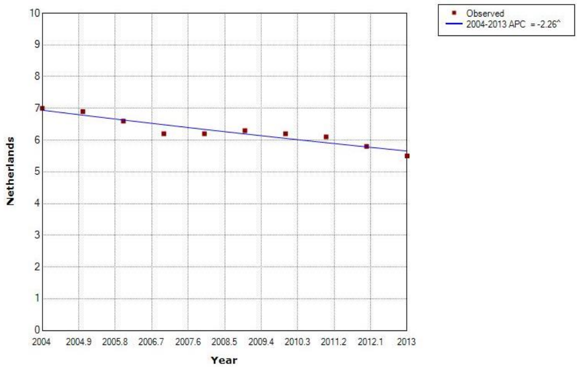

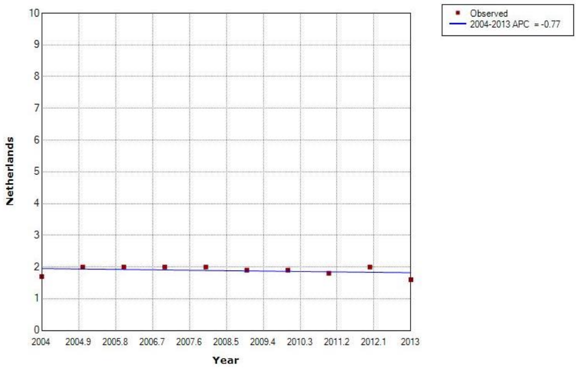

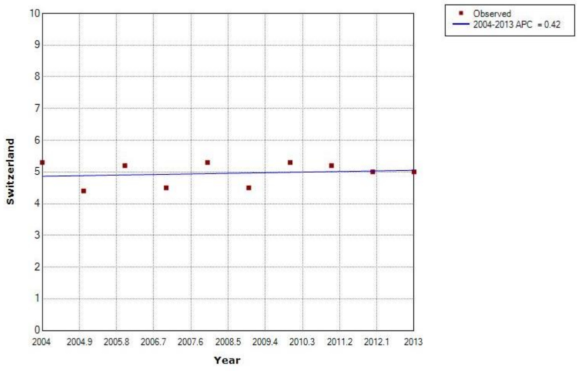

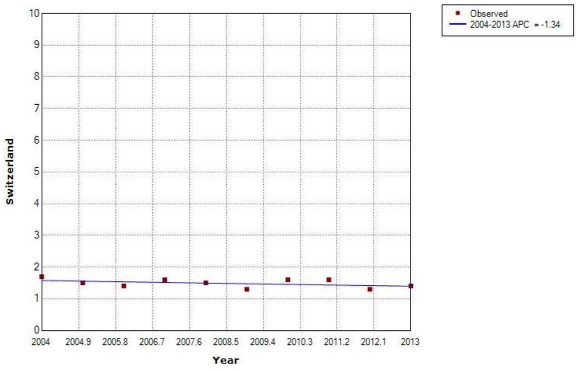

7) Southern Europe

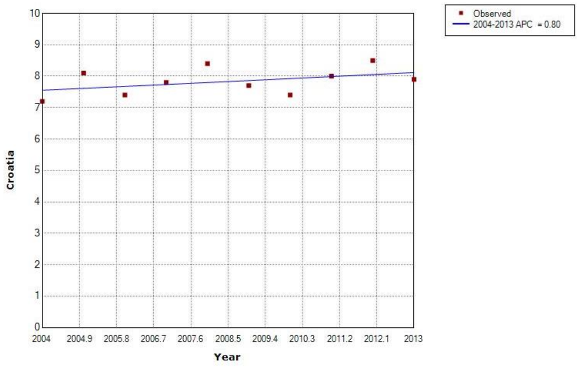

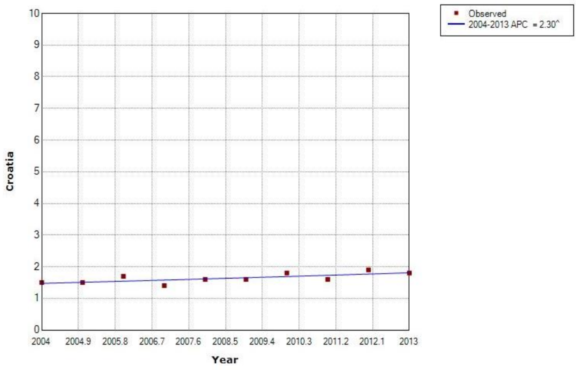

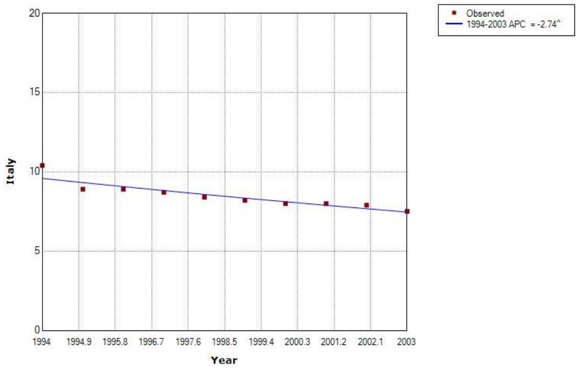

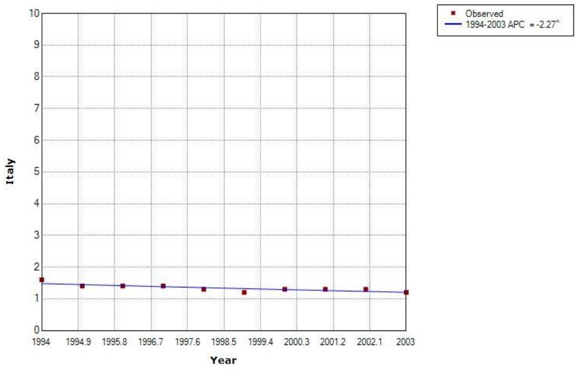

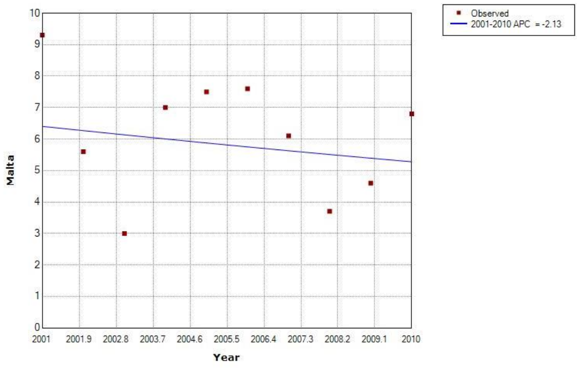

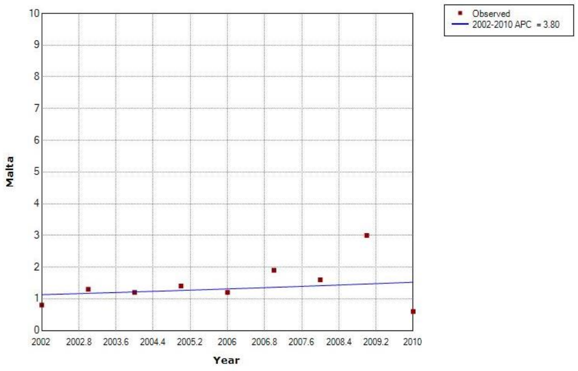

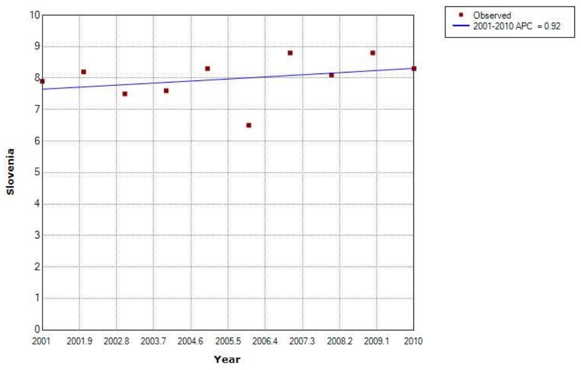

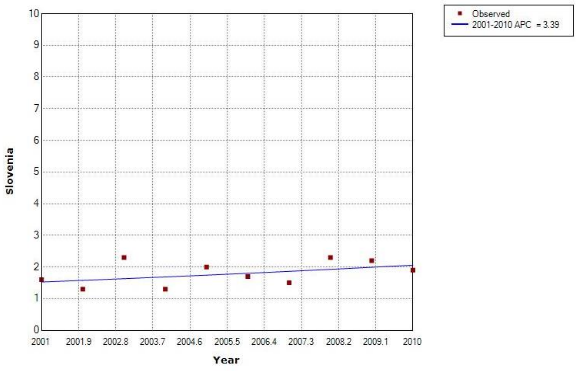

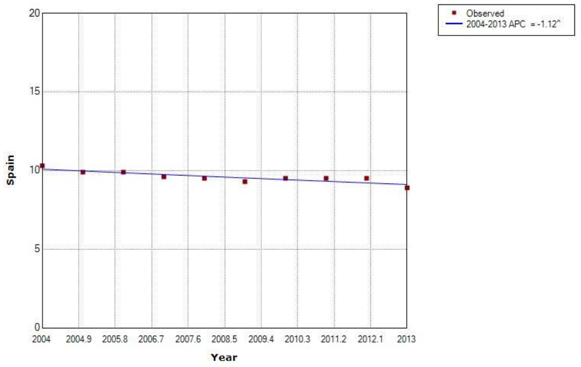

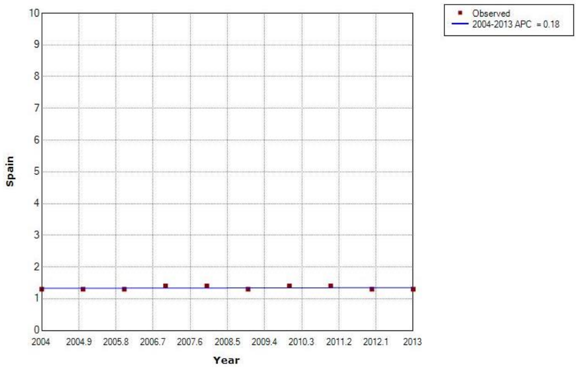

8) Eastern Europe

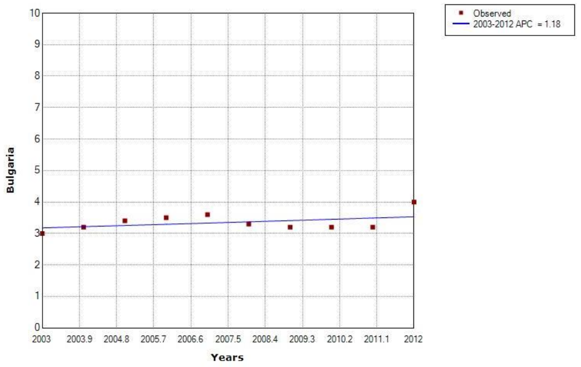

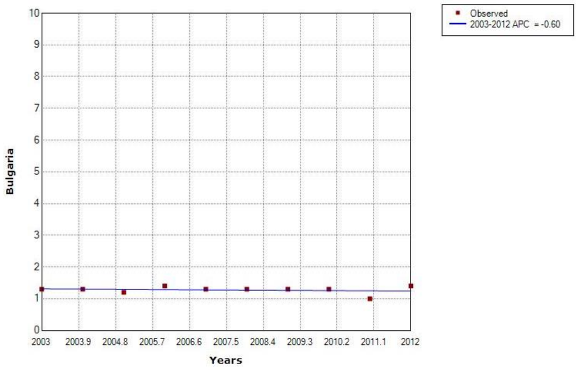

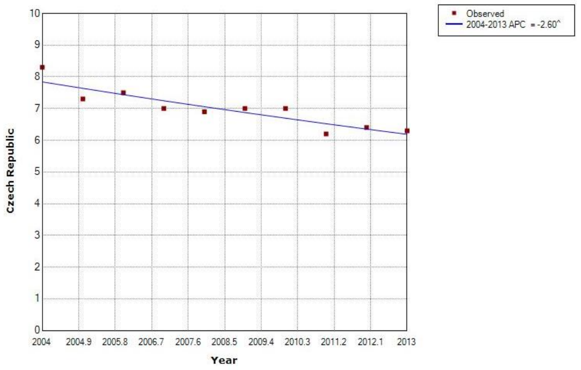

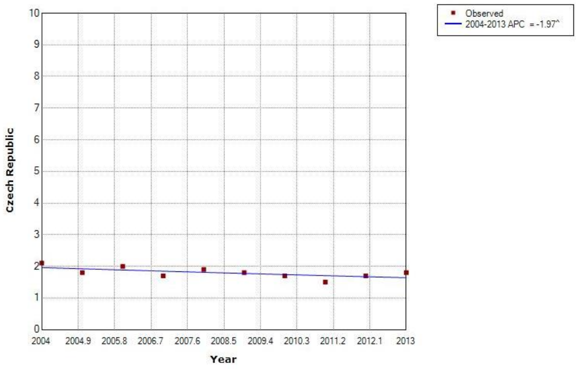

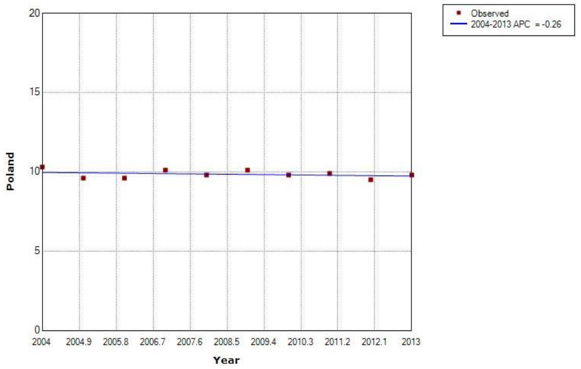
Observed
2004-2013 APC = -0.26
Poland
Year

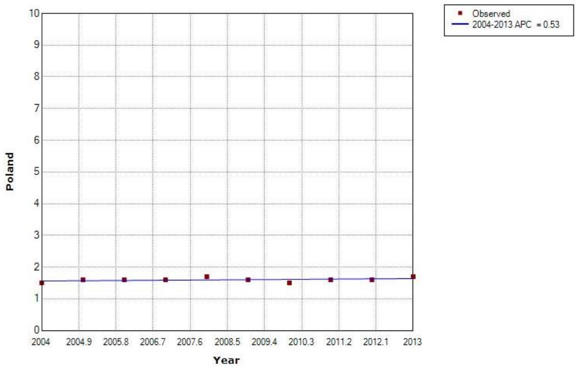
Observed
2004-2013 APC = 0.53
Poland
Year

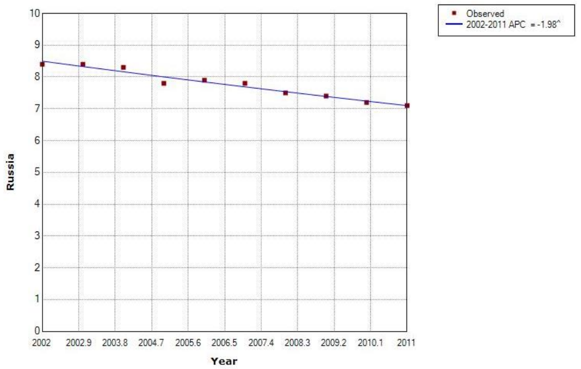
Observed
2002-2011 APC = -1.98^
Russia
Year

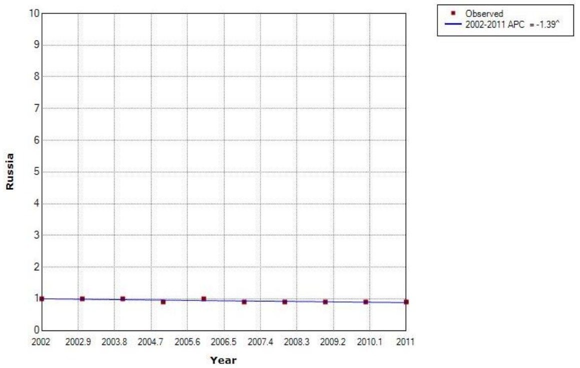
Observed
2002-2011 APC = -1.39^
Russia
Year

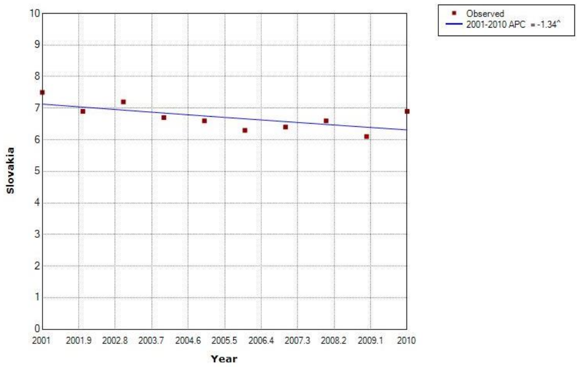
Observed
2001-2010 APC = -1.34^
Slovakia
Year

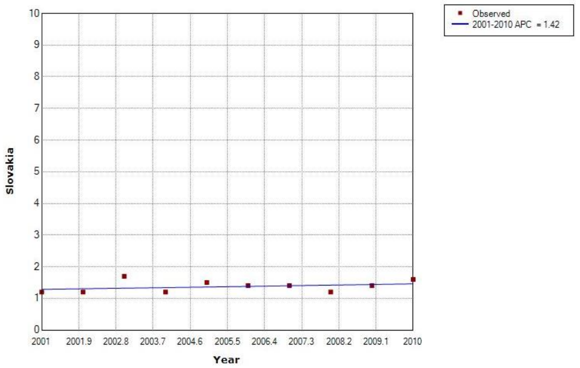
Observed
2001-2010 APC = 1.42
Slovakia
Year
